# Glass Transition and Yielding of Ultrasoft Charged Spherical Micelles

**DOI:** 10.1021/acs.macromol.4c03215

**Published:** 2025-04-30

**Authors:** Roshan Akdar Mohamed Yunus, Utku Gürel, Aleksander Guzik, Philippe Dieudonné-George, Marc C.A. Stuart, Christos N. Likos, Patrizio Raffa, Domenico Truzzolillo, Andrea Giuntoli, Daniele Parisi

**Affiliations:** 1 Department of Chemical Engineering, Engineering and Technology Institute Groningen, 3647 University of Groningen, Nijenborgh 3, Groningen 9747 AG, The Netherlands; 2 Zernike Institute for Advanced Materials Micromechanics, 3647 University of Groningen, Nijenborgh 3, Groningen 9747 AG, The Netherlands; 3 Laboratoire Charles Coulomb (L2C), UMR 5221 CNRS Université de Montpellier, Montpellier 34095, France; 4 Electron Microscopy, Groningen Biomolecular Sciences and Biotechnology Institute, 3647 University of Groningen, Nijenborgh 7, Groningen 9747 AG, The Netherlands; 5 Faculty of Physics, 27258 University of Vienna, Boltzmanngasse 5, Vienna 1090, Austria

## Abstract

Colloids are widely used as model systems to study the glass transition, with much research focused on hard or soft neutral colloids and their mixtures. However, the glass transition of soft-charged spherical colloids and its dynamic effects remain underexplored. This study assesses the glass transition of polymeric multiarm charged soft colloids using shear rheology, coarse-grained molecular dynamics (MD) simulations, and X-ray scattering. Strong particle–particle correlations, driven by electrostatic interarm repulsion, inhibit Newtonian behavior in the dilute regime. The liquid-to-glass transition occurs at just 0.25 wt %, marked by weak caging and minimal interdigitation of arms, as evidenced by particle contact analysis in MD simulations and the strong frequency dependence of the dynamic moduli. Particle shells interdigitate only well within the glassy regime, leading to a nearly frequency-independent rheological response. The weak volume fraction dependence of the yield stress sets these charged systems among the softest colloids reported in the literature.

## Graphical abstract

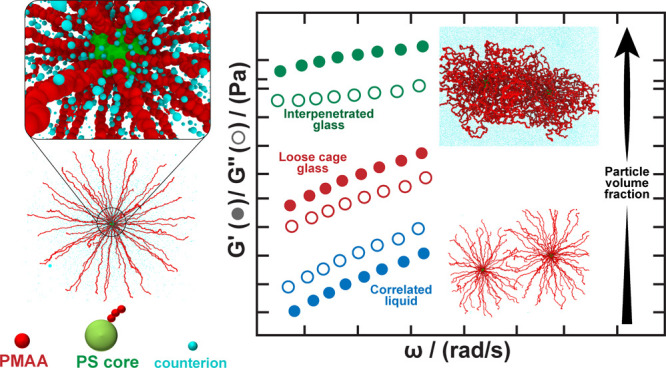

## Introduction

The glass transition has been extensively explored by researchers in the present era of condensed matter physics. In this respect, colloidal particles serve as ideal glass formers in the realm of this research.^1,2^ When particle interactions are driven by volume exclusion, glass-like properties mainly arise from particle packing. As the packing fraction increases, the intensified crowding slows down particle relaxation, leading to significant rheological changes. A colloidal suspension initially exhibits an almost negligible elastic response. However, as particle concentration increases, the system transitions to a viscoelastic state and eventually displays predominantly elastic behavior, with the storage modulus dominating its shear response. Thus, rheological measurements have proven to be an excellent tool for investigating the glass transition in various densely packed systems.^3,4^

Many pioneering experimental and theoretical studies have been dedicated to understanding the glass transition for hard sphere suspensions, which have served as a reference system for a long time.^5–7^ Here, when the particle volume fraction exceeds 0.58, the colloids cannot move past each other, quenching into a glassy state characterized by caged diffusion and yield-stress behavior.^8^ However, the surge of caging occurs over a thin span of concentrations.^4^ Interestingly, soft colloidal particles, like grafted nanoparticles, emulsions, microgels, multiarm star polymers, and copolymer micelles, can deform or overlap partially by interdigitation, thus enabling them to be crammed at even higher packing fractions, allowing to study the glass state both rheologically and microscopically more in-depth.^9–19^ Additionally, unlike hard spheres, these softer systems exhibit a gradual transition to their glassy regime.^20^

Soft colloids, such as hairy particles or star-shaped aggregates with multiple polymer chains attached to a common core, form an intriguing class of macromolecular assemblies in condensed matter physics. These structures bridge the gap between linear polymer chains and spherical colloids.^21^ Their polymeric corona with its ability to interpenetrate, deform, and deswell confers an interesting phase behavior to their crowded suspensions.^14^ Still, despite the current active research involving hairy nanoparticles, the role played by the charge at their periphery, which gives them the name of *polyelectrolyte stars*, stays partially unexplored. One such system will serve as the crux of the present work. It is well established that in hard and charged colloids, particles interacting through long-range Coulombic forces, form a low-density solid phase known as a “Wigner glass”.^2,22–42^ The most notable of the studied systems is the charged suspension of Laponite particles. However, a recent commentary by Y. M. Joshi et al.^43^ critically refuted the occurrence of Wigner glassy states in Laponite dispersions, providing strong evidence of sol–gel transitions instead. However, the nature of the laponite plays a crucial role in particle–particle interactions. Nonetheless, the effect of the additional electrostatic repulsion in charged hairy colloids on the glass transition and rheology has not been systematically addressed.

Over the years, the conformational behavior of polyelectrolyte stars has always been of interest. Some of the earliest studies have dealt with the development of scaling theories for such systems. The theoretical works of Borisov and co-workers^44–51^ predominantly focused on scaling laws involving the effect of ionic strength, polymer concentration, number of arms, and effect of salt. Shusharina and Rubinstein^52^ performed an extensive investigation, leveraging scaling arguments, to map the conformational phase diagram of polyelectrolyte stars, while Jusufi et al.^21^ and Likos et al.^53^ further studied these charged systems with simulations and theory.^54,55^ It is worth noting that among these seminal works, the effect of concentration was solely discussed theoretically by Shusharina and Rubinstein^52^ and Borisov and co-workers.^45^ Our work bridges the gap between experiments and theory, offering a fresh perspective on the dense suspension of soft-charged systems, which have seen only limited experimental investigation so far.^56–60^

The interactions between charged particles formed from the diblock copolymer poly­(styrene-*block*-acrylic acid) in water have been investigated via small-angle neutron scattering (SANS) experiments by Korobko et al.^57^ The authors reported that when the arms are fully charged (under no-salt conditions), upon increasing the concentration, the particles shrink to accommodate additional particles and engage in interdigitation once their packing fraction crosses 0.53. In a follow-up study,^59^ they showed that, for fully charged particles (under no-salt conditions), the counterions were strongly adsorbed in the interior of the particle, giving rise to osmotic pressure, and leading to significant stretching of the arms. Interestingly, they also presented the frequency response of particles with 50% of their outer corona being charged. The moduli values G’ and G” exhibited a strong frequency dependence (with G’ > G”) and suggested that they formed an interconnected, physical gel. Furthermore, P. Matějíček et al.^60^ in their work on polystyrene-*block*-poly­(methacrylic acid), PS–PMA micelles (with the PMA block being charged) reported that the corona was charged and that unscreened electrostatic forces acted at fairly long distances and dominated the solution behavior. The team’s light scattering data reported strong interparticle interactions, highlighting the complex dynamics within these systems. However, a comprehensive understanding of how the glass transition manifests in soft-charged colloids remains elusive, leaving many questions unanswered. For instance, how does the charged corona of these soft particles influence their liquid-to-glass transition? How does increasing concentration impact the interpenetration between particles, and what effects does this have on their rheological properties under shear? Additionally, to what extent does the charged corona contribute to the softness of the system? Addressing these questions requires a detailed comparison of these vitrified systems with other well-known colloidal glasses, both hard and soft.

In this study, we used 4-armed diblock star polystyrene (PS)—polymethacrylic acid (PMAA) polyelectrolytes, which assemble into micelles in water, as a model system for ultrasoft charged particles. We first present the characterization of the system performed via dynamic light scattering (DLS), small and wide X-ray scattering, and cryo-transmission electron microscopy (Cryo-TEM). We utilized small-amplitude oscillatory shear rheology to map the dynamic state diagram of such systems, and clearly showcase the change in dynamics and state behavior, across vitrification and deep into their glassy regime. Additionally, to corroborate our rheological findings, we present computational results using coarse-grained molecular dynamics simulations (MDS). We simulated charged systems ranging from dilute to concentrated regimes, complementing our rheological data. Finally, start up of shear rate experiments were performed to investigate the behavior of these particles under continuous flow conditions and assess their yielding behavior. We compare it to other state-of-the-art systems and show how the presence of charges and their molecular architecture make them ultrasoft colloidal particles with remarkable features.

## Materials and Methods

### Multiarmed Polyelectrolyte Particles

The system investigated in this research is a multiarmed micellar polyelectrolyte particle. It is formed from an amphiphilic diblock star-shaped copolymer, comprising a polystyrene (PS) core with four polymethylarcylic acid (PMAA) arms. The chemical molar mass for PS and PMAA are 104 g/mol and 86 g/mol respectively, and the number of monomers per arm is 23 and 367,^61^ respectively. Thus, the molar mass of an individual 4-arm star is 3.4·10^4^ g/mol. Due to the hydrophobic nature of PS, the diblock star polymer in the presence of a water medium collapses, and self-assembles to generate micellar architectures, whose core is composed of PS and stretched PMAA arms in the corona. A 1.0 wt % diblock star polyelectrolyte stock solution was prepared through radical controlled polymerization technique (RAFT) as per the protocol established by Raffa et al.^61^ Solutions of lower concentrations were obtained by diluting aliquots of 1.0 wt % solution with Milli-Q water and higher concentrations were achieved by evaporating the water from the stock solution. In this study, concentrations varying between 0.1 – 3.0 wt % were investigated.

### Dynamic Light Scattering

The size of the micelle was estimated by quasi-elastic light scattering (QELS) technique. It was carried out with a Zetasizer Ultra Malvern Instrument equipped with a He–Ne laser (λ = 633 nm). The measurement was carried out at 20 °C and detection angles of θ = 12.78° and 174.7°. Each angle corresponds to *q*, the scattering wave vector which is also a function of the refractive index of the solvent, i.e. water (*n*_*solvent*_ = 1.33), and the wavelength of the laser beam, λ (= 633 nm):

$$q=\frac{4\pi n_{solvent}}{\lambda }\sin\left(\frac{\theta }{2}\right)$$ i

The data obtained were fitted with a single exponential function and for each angle, a decay rate (Γ; inverse of relaxation time τ), and consequently, diffusion coefficient, D, can be extracted:

$$\Gamma =Dq^{2}$$ ii

*D* is related to the hydrodynamic radius *R*_H_ of the micelle, and it could be calculated via the Stokes–Einstein-Sutherland equation:

$$D=\frac{k_{B}T}{6\pi \eta_{solvent}R_{H}}$$ iii

with *k*_*B*_ is the Boltzmann constant, *T* is the absolute temperature, and the η_*solvent*_ = 0.91 mPa.s is the viscosity of the solvent.

### Cryo Transmission Electron Microscopy (Cryo-TEM)

The core of the micelle was imaged with cryo-TEM. An aliquot of 1.0 wt % diblock star polyelectrolyte solution was diluted and deposited on previously glow-discharged, holey carbon-coated grids (3.5/1 Quantifoil Micro Tools, Jena, Germany). The excess liquid was blotted for 11 s, and the grids were vitrified in liquid ethane with a Vitrobot (FEI, Eindhoven, The Netherlands).

Subsequently, the samples were observed with a FEI Tecnai T20 electron microscope equipped with a Gatan model 626 cryo-stage operated at 200 keV. Both the TEM micrographs were recorded under low-dose conditions with a slow-scan CCD camera.

### Rheology

The rheological measurements were performed with a Discovery Hybrid Rheometer (HR-2) from TA Instruments (United States). All the measurements were carried out at 20 °C with a parallel plate configuration, and based on the viscosity of the samples, 25- or 40 mm diameter plates were utilized. It was ensured that the sample remained in a water-saturated atmosphere to hinder water evaporation.

The diblock star polyelectrolyte solutions were loaded into the rheometer, and they were subjected to rejuvenation and aging (Figures S5–S10). The rejuvenation is a dynamic time sweep (DtS) at 1 rad/s and a selected 200% strain (ensured to choose a strain falling in the nonlinear regime by performing a DSS at 1 rad/s), typically for 60 s – 100 s (until steady state was observed) to erase the mechanical history of the sample. Subsequently, the aging is a DtS conducted at 1 rad/s and strain falling in the linear viscoelastic regime for 200 s to build up the internal stress-free structure of the material. Consequently, a small amplitude oscillatory shear (SAOS) technique was employed to probe the linear viscoelastic (LVE) response of the sample. Dynamic strain sweeps (DSS) were executed at 100 rad/s to determine a strain amplitude within the LVE regime. Frequency sweeps were performed over a range of frequencies varying from 100 to 0.01 rad/s.

The start-up of shear experiments were executed for samples exhibiting solid-like response in the LVE spectrum. Shear rates varying from 0.01 s^–1^ to 100 s^–1^ were applied to the samples in a well-defined protocol. The multiarmed polyelectrolyte solutions were loaded into the rheometer and prior to applying each shear rate, they were subjected to rejuvenation and aging. Consequently, the sample was subjected to steady shear until steady state values were obtained. The determined flow curves were corrected by applying the Weissenberg-Rabinowitsch correction factor^62^ to account for the radial shear rate dependence in the parallel plate geometry:

$$\mathrm{\gamma ̇}={\mathrm{\gamma ̇}}_{app}\left[\frac{1}{4}\left(3+\frac{d\;\ln\;{\mathrm{\gamma ̇}}_{app}}{d\;\ln\;\sigma }\right)\right]$$ iv

where γ̇ is the corrected true shear rate, γ̇*_app_* is the apparent shear rate, and σ is the shear stress.

### Molecular Dynamics Simulations

#### Micelle Model

Using a coarse-grained bead–spring model, we represent an entire micelle with long PMAA flexible chains grafted onto the PS core, which is represented as a single large bead.^63^ We follow the assumption taken from the cryo-TEM data that each micelle is composed of 13 aggregated star polymers, with ∼ 4.7 nm core for the PS radius. Each micelle has 13*4 = 52 PMAA arms of 183 beads, where each bead corresponds to one PMAA Kuhn length (roughly 2 PMAA monomers) and has a diameter of approximately σ = 1 *nm*, which we consider as the fundamental length scale of the simulation. The PS core has a radius R = 4.7σ ∼ 4.7 *nm*. Each simulation contains 64 micelles, with a total of *N*_*PMAA*_ = 64*52*183 = 609024 PMAA beads, and *N*_*ion*_ = *N*_*PMAA*_ separate beads to represent counterions and preserve charge neutrality. The solvent is treated implicitly.

Nonbonded beads at distance *r* interact with a truncated and shifted Lennard-Jones potential:

$$U_{nb}=4\varepsilon \left[{\left(\frac{\sigma }{r-\Delta }\right)}^{12}-{\left(\frac{\sigma }{r-\Delta }\right)}^{6}\right];r<r_{c}+\Delta$$ v

where ε is the energy scale of the system (equal for all interactions). *r*_*c*_ = 1.122σ is the cutoff used to include only repulsive interactions (representing good solvent conditions), while only for PS–PS cores *r*_*c*_ = 2.5σ. Δ is a shift factor to account for particles of different sizes. Δ = 0, except for the interactions of the PS core, where Δ_*PS* – *PS*_ = 8.4, Δ_*PS* – *PMAA*_ = Δ_*PS* – *ion*_ = 4.2. Bonded beads interact with a harmonic potential *U*_*b*_ = *k*(*r* – *r*_0_)^2^, with stiffness *k* = 2500ε/σ^2^ and resting length *r*_0_ = 1.0σ for PMAA–PMAA bonds, and *r*_0_ = 5.2σ for PS–PMAA bonds.

Charged beads interact with a Coulomb potential $U_{coul}=\varepsilon \frac{q_{i}q_{j}}{\epsilon r}$ with a cutoff of *r*_*c*_*coul*_ = 10σ, where *q*_*PMAA*_ = – 1; *q*_*ion*_ = +1, ε is the energy scale of the system, ϵ = 1 is the dielectric constant, and the long-range Coulomb interactions are evaluated with a PPPM method with an estimation accuracy of 10^–4^.^64^ The energy scale ε can roughly be mapped to a few *kJ*/*mol* (e.g., 4.1 *kJ*/*mol* for polystyrene^65^), though the dynamics of our simulations is greatly accelerated by the coarse-graining model and the implicit treatment of the solvent.

We take the mass of one PMAA bead *m*_*PMAA*_ = *m* as the unit mass of our system. *m*_*ion*_ = *m* for simplicity, while for the large PS core *M*_*PS*_ = 691*m*, assuming 13*4*23 = 1196 PS monomers in the micelle core, and the relative molecular weight of PS and PMAA. All quantities can be expressed in the reduced Lennard-Jones units ε, σ, *m* and the derived time unit $\tau =\sqrt{\frac{m\sigma^{2}}{\varepsilon }}$ .

#### System Preparation, Equilibration, and Data Production

Micelles are randomly generated in a large simulation box, then counterions are generated in number equal to the PMAA beads. Charge interactions are initially turned off, simulating the limit of high-salt concentration. The volume of the simulation box is chosen so that a range of polymer concentration in volume percentage *c* (*vol* %) = {10^–3^ – 3.0} is investigated, similar to the experimental range. A time step of Δ*t* = 0.005τ is used. A soft repulsion is initially used for nonbonded interactions to avoid bad dynamics, then the systems are equilibrated at temperature *T* = 1 with a Langevin thermostat for 12500τ, which is enough to fully relax the system due to the low density, meaning that we observe full decorrelation of the end-to-end vector of the micelle arms.

The desalting process is mimicked by gradually turning on the charges from zero to the full Coulomb interaction in a time τ_*charge*_ = 500τ, then relaxing the system for another 500τ. In this phase, the long PMAA arms greatly expand because of the Coulomb repulsions, and the equilibration time is only sufficient for the local relaxation of polymer chains, not for the rearrangement of entire micelles. Since we end up in a jammed state (see results and scaled-down model in the SI), we expect the time scales for full relaxation to be in any case inaccessible to our simulations, and the best we can do to mitigate this effect is to average over multiple replicas. To this end, 8 independent systems are simulated starting from randomized initial positions and velocities of all beads, with the equilibration of the neutral micelles further ensuring statistical independence. For all our simulation results, the error bars coming from the variance among these replicas are smaller than the symbol size, showing that the results of these simulations are robust.

The final data production is done after this step over a time 250τ, and averaged over 8 independent replicas of the system. The baseline comparison with neutral systems is performed by skipping the charging step and moving to the production phase without ever turning on the Coulomb interactions.

#### Contact Analysis

We quantify the number of interstar contacts by calculating the distance matrix for all pairs of stars. A contact is defined if the distance between the centers of two-star beads is less than 2.5σ. For a star consisting of N beads, a complete contact with another star of the same size would result in N^2^ contacts. To determine the average number of interstar contacts, we normalize the total number of contacts by the number of stars in the system (64), resulting in the contacts per star at each concentration value.

### X-ray Scattering

SAXS/WAXS measurements were conducted using an in-house setup located in Laboratoire Charles Coulomb, University of Montpellier. The system featured a high-brightness, low-power X-ray tube paired with an aspheric multilayer optic, specifically the GeniX^3D^ from Xenocs, which provided an ultralow divergent X-ray beam (0.5 mrad, λ = 0.15418 nm). To achieve a clean 0.6 mm beam diameter and a flux of 35 million photons per second at the sample, scatterless slits were employed. The experiment was performed in a transmission configuration, and scattered intensity was captured by a 2D ″Pilatus″ detector by Dectris (490 × 600 pixels) with a pixel size (area) of 172 × 172 μm^2^. The detector was positioned 1.9/0.2 m from the sample, which was loaded into cylindrical quartz capillary tubes with a 1 mm diameter. SAXS/WAXS data were collected across a scattering wavevector range of 0.05 nm^–1^ to 20 nm^–1^. The temperature was maintained at a constant room temperature (T = 22.0 ± 0.5 °C). All recorded intensities were corrected for transmission, and the contribution from the empty cell was subtracted.

The collected intensities were fitted to eq 5 where *P*_*Mic*_(*q*) is the Pedersen’s micelle form factor that we report explicitly here^66,67^

$$P_{Mic}\left(q\right)=p^{2}\rho_{core}^{2}F_{s}\left(q,R_{core,X}\right)+p\rho_{shell}^{2}F_{c}\left(q,R_{shell,X}\right)+p\left(p-1\right)\rho_{shell}^{2}F_{cc}\left(q,R_{core,X},R_{shell,X}\right)+2p^{2}\rho_{shell}\rho_{core}F_{sc}\left(q,R_{core,X},R_{shell,X}\right)$$ vi

The first term on the r.h.s. of eq [Disp-formula eqvi] is written as *F*_*s*_(*q*, *R*_*core*, X_) = Φ­(*qR*_*core*, X_)^2^ with Φ­(*x*) = 3*x* ^–3^(*sin* (*x*) – *xcos*(*x*)), if one assumes a constant density core of radius *R*_*core*_. An approximate expression for the chains in the corona can be written by considering Gaussian chains: *F*_*c*_(*qR*_*shell*, *X*_) = *g*(*q* ^2^ *R*_*shell*, *X*_^2^/6) with *g*(*x*) = 2*x* ^–2^(*e* ^–*x*^ – 1 + *x*). With these approximations the cross-correlation terms are given by *F*_*cc*_(*q*, *R*_*core*, X_, *R*_*shell*, *X*_) = Ψ­(*qR*_*shell*, *X*_)^2^χ­(*qR*_*core*, X_)^2^ with Ψ­(*x*) = *x* ^–1^(1 – *e* ^–*x*^) and χ­(*x*) = *x* ^–1^ *sin* (*x*); *F*_*sc*_(*q*, *R*_*core*, X_, *R*_*shell*, *X*_) = Ψ­(*qR*_*shell*, *X*_)­Φ­(*qR*_*core*, X_)­χ­(*qR*_*core*, X_)

## Results and Discussion

### Characterization and Molecular Modeling

The multiarmed polyelectrolyte soft colloids investigated in this study are obtained from the self-assembly of 4-arm star polymers in salt-free water. Each arm consists of *N*_*PS*_ = 23 monomers of polystyrene (PS) (inner block) and *N*_*PMAA*_ = 367 monomers of negatively charged polymethacrylic acid (PMAA) (outer block).^61^

Dynamic light scattering (DLS) in the dilute regime (0.001 wt %) showed single exponential decay autocorrelation functions at two different scattering angles (174.7° and 12.78°) as shown in Figure 1a, allowing for the determination of the hydrodynamic radius *R*_*H*_ of the polyelectrolyte micelle to be 186 nm ± 14 nm (see Materials and Methods section). By combining this result with Cryo-TEM and molecular modeling it is possible to estimate the size of the PS core, the aggregation number (*N*_*agg*_), namely the number of diblock 4-arm stars per micelle, and the size of the charged shell.

**Figure 1:**
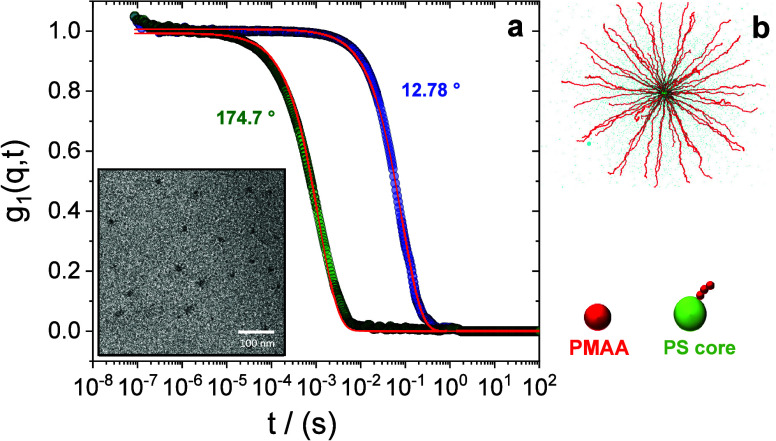
(a) Electric field autocorrelation functions g_1_(q,t) at scattering angles 174.7° and 12.78° for multiarmed PS–PMAA polyelectrolyte solutions at 0.001 wt % and 20 °C. Red lines are the best fit for the data to a single exponential function. The inset represents a cryo-TEM micrograph at *c* = 0.2 wt % depicting the PS core of the multiarmed PS–PMAA polyelectrolyte solutions. (b) The experiments are supported by MD simulations of a bead–spring model where the assembly of the PS monomers of 13 stars is represented with a single rigid core, with grafted chains where one bead corresponds to one PMAA Kuhn length.

The chemical molar mass for PS is 104 g/mol, making the total molar mass along one arm equal to 23·104 = 2392 g/mol. The Kuhn molar mass for PS is 720 g/mol,^68^ thus, there are 2392/720 = 3.3 Kuhn monomers per PS arm. PS is also strongly hydrophobic, and it is therefore expected that the PS blocks in the 4-arm star fully collapse in water. According to the Daoud and Cotton model, the radius of a fully collapsed star can be expressed as^69^

$$R={\left(fN\right)}^{1/3}\frac{b}{2}$$ 1

where *f* is the functionality (number of arms, 4 in this case), *N* is the Kuhn degree of polymerization, and *b* (= 1.8 nm^68^) is the Kuhn length.

Considering that only the PS collapses in water, eq 1 yielded a value of 2 nm. The inset of Figure 1a depicts a cryo-TEM micrograph of a diluted multiarmed polyelectrolyte solution, where the PS core of the micelle is discernible (dark spots in the inset of Figure 1a). Analysis of the micrograph with the ImageJ software yielded a radius of the core of the micelle, *R*_*core*_, to be 4.7 ± 0.4 nm. As a result, the number of 4-arm stars per micelle, or aggregation number *N*_*agg*_, can be determined as the ratio between the total volume of the core of a micelle and the volume of the PS core in a single star:

$$N_{agg}=\frac{{R_{core}}^{3}}{R^{3}}$$ 2

Equation 2 revealed that on average 13 individual diblock star polymers (*N*_*agg*_ = 13) are comprised into a single micelle, yielding a total of 52 arms per micelle. Similar numbers were also attained in a previous work.^70^ Note that due to the high glass transition temperature of the PS core, the aggregation number remains fixed without any unimer exchange.^59^ These estimates were used to calibrate the architecture of the model micelle (Figure 1b) used in the molecular dynamics simulations. The MAA monomer possesses a length, *l*, equal to 0.5 nm.^47^ In the absence of salt, the PMAA arms are significantly stretched, therefore, it is possible to estimate the length of the PMAA arm, *R*_*PMAA*_, as *N*_*PMAA*_ *l* = 183.5 nm. The total radius of the diblock star polymer micelle is, therefore, *R*_*tot*_= *R*_*core*_ + *R*_*PMAA*_ = 4.7 + 183.5 = 188.2 nm. Remarkably, the obtained value is in very good agreement with the hydrodynamic radius (*R*_*H*_) obtained by means of DLS experiments (186 ± 14 nm), making the theoretical estimates quite robust.

Owing to the softness of the system, an effective volume fraction ϕ_eff_ can be estimated as^71,72^

$$\phi_{eff}=\frac{c}{c^{*}}$$ 3

It is important to note here that the overlap concentration marks the concentration at which the transition between the dilute and the semidilute regime occurs, i.e. when the particle self-diffusion becomes more sensitive to an increase in concentration, but it does not necessarily reflect physical overlap between particles, as shown by Loppinet et al.^73^ The overlap concentration is commonly estimated as^71,72^

$$c^{*}=\frac{M_{micelle}}{\frac{4}{3}\pi {R_{H}}^{3}N_{A}}$$ 4

where *M*_*micelle*_ is the total molar mass of a single micelle, *N*_*A*_ Avogardo’s number, and *R*_*H*_ the hydrodynamic radius determined via light scattering. *M*_*micelle*_ is determined as follows. The chemical molar mass for PS and PMAA are 104 g/mol and 86 g/mol respectively, and the number of monomers per arm is 23 and 367, respectively. Thus, the molar mass of a single 4-arm star is 3.4·10^4^ g/mol and the number of stars per micelle is 13 (*N*_*agg*_), the total molar mass of a single micelle *M*_*micelle*_, is 1.76·10^6^ g/mol. Hence, eq 4 yields c* = 0.011 wt %.

The effective volume fractions ϕ_eff_ used in this work are reported in Table 1.

**Table 1:** Effective Volume Fractions of Different Diblock Star Polyelectrolyte Solutions Used in This Work: Concentration of the Solution, *c*, and the Effective Volume Fraction, ϕ_eff_

*c*	0.1 wt %	0.2 wt %	0.25 wt %	0.3 wt %	0.5 wt %	1 wt %	2 wt %	3 wt %
ϕ_eff_	9	18	23	27	45	91	182	273

The inner structure of the multiarmed polyelectrolyte particles was assessed through small-angle (SAXS) X-ray scattering in the concentration range 0.2 wt % ≤ *c* ≤ 3 wt % and wide-angle (WAXS) X-ray scattering at *c* ≥ 1 wt % (Figure 2a). At particle concentrations below 1 wt %, the scattering signal of the samples in the WAXS region was too weak to be resolved.

**Figure 2:**
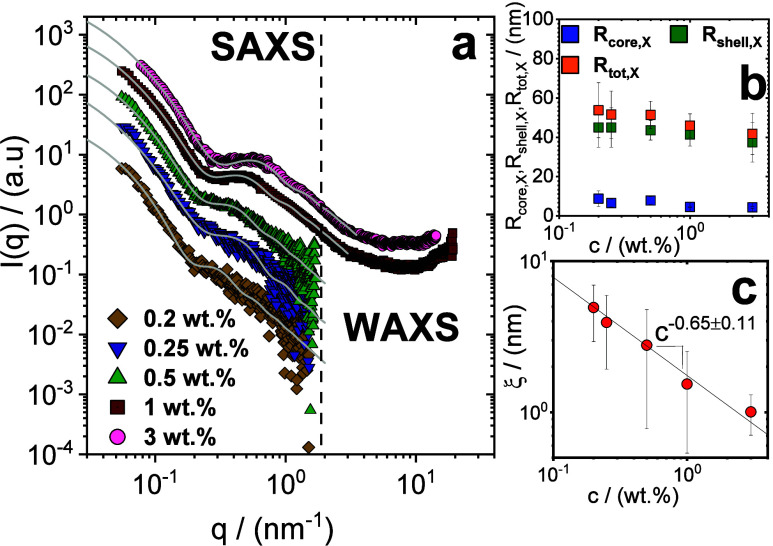
(a) Combination of small-angle and wide-angle scattering profiles (SAXS/WAXS) as a function of the scattering vector *q* for the multiarmed PS–PMAA polyelectrolyte solutions across various concentrations (see legend). The gray lines are the best fits to the micelle form factor (eq 5) as detailed in the text. The core radius *R*_*core,X*_, the shell radius *R*_*shell,X*_, the total micelle radius *R*_*tot,X*_ = *R*_*core,X*_ + *R*_*shell,X*_ and the correlation length ξ obtained from the fits are shown in panels (b) and (c).

The intensity profiles I­(q) are characterized by the evident onset of a shoulder that appears at *q* ≈ 0.05 nm^–1^ and becomes prominent upon increasing *c*. Previous X-ray scattering studies on sodium-sulfonated polystyrene (NaPSS) star-branched polyelectrolytes^74^ have pointed out that a peak structure develops progressively as *c* increases from the dilute regime (c < c*), indicating some ordering phenomenon. In that work, the observed maximum sharpens with increasing concentration, its height increases and its position in *q* scales as *c* ^1*/*3^, following therefore the geometric scaling.^74^ By contrast, in the present case, the shoulder position does not remarkably shift with concentration and therefore we attribute this feature to single-particle scattering. To corroborate this hypothesis, we fit the entire set of data with Pedersen’s form factor model for polymeric micelles^66,67^ with an extra contribution at high-*q* vectors related to fluctuations originating from chain statistics and the interchain interactions of PMAA chains inside the shell.^75^ The intracorona contribution, due to the overlapping crumpled arms (in the semidilute regime), is well reproduced by an Ornstein–Zernike (OZ) type term and the entire form factor reads as

$$I\left(q\right)\propto P\left(q\right)=P_{Mic}\left(q,R_{core,X},R_{shell,X},N_{agg},\rho_{core},\rho_{shell}\right)+\frac{\alpha }{1+\xi^{2}q^{2}}$$ 5

where the form factor model for polymeric micelles *P*_*Mic*_(*q*, *R*_*core*, *X*_, *R*_*shell*, *X*_, *N_agg_*, ρ_*core*_, ρ_*shell*_) is a function of the core radius *R*_*core*, *X*_, the gyration radius of the chains in the shell *R*_*shell*, *X*_, the micelle aggregation number *N*_*agg*_ and the excess scattering lengths ρ_*core*_ and ρ_*shell*_ of the core and the outer chains (see Materials and Methods for details). The OZ term ξ accounts for the length scale of density fluctuations inside the corona (order of 1 nm), while α quantifies the relative amplitude of the OZ contribution.

Such a model captured quite well the observed data, as shown in Figure 2a. The fitting curves are very sensitive to *R*_*core*, *X*_, *R*_*shell*, *X*_, and ξ, and they provide a robust estimation of the shell size, the core size, and the length scale of density fluctuation in the present crowded suspensions of polyelectrolyte stars. By contrast, the fit is poorly affected by large variations (over 2 orders of magnitude) of the aggregation number *N*_*agg*_ and the ratio $\frac{\rho_{core}}{\rho_{shell}}$ whose expected values (*N*_*agg*_ = 13, ρ_*shell*_/ρ_*core*_ = 0.8) are within the large standard errors obtained by the nonlinear regression of the data (more details in the Materials and Methods section). Figure 2b shows the concentration dependence of *R*_*core*, *X*_, *R*_*shell*, *X*_, *R*_*tot*, *X*_, and ξ obtained from the best fit of the scattering intensity profiles. We find that the radius of the core changes slightly with increasing concentration, whereas the shell shrinks from ∼ 45 nm to ∼ 37 nm from the viscoelastic liquid (*c* < 0.24 wt %), albeit well above the overlap concentration, to the glassy regime, owing to the change in the conformation of the arms with increasing particle content.

A decrease in the correlation length ξ with increasing particle concentration was found to follow a power-law decay ξ*(c) ∼ c* ^–α^ (Figure 2c). We find α = 0.65 ± 0.11 that gives a decay compatible with the scaling ξ*(c) ∼ c* ^*–0.5*^ predicted for the correlation length in semidilute solutions of linear polyelectrolytes with condensed and free counterions, and valid also for polyelectrolyte stars in the semidilute regime.^52^ The absence of any distinct Bragg peaks across the concentrations explored, points to a homogeneous distribution of particles in line with the presence of an amorphous structure. Moreover, the absence of clear structure peaks in the accessible range of scattering wavevectors is in line with the expected minimum average distance between the micelles obtained from MD simulations (see Figure 6a) that can be obtained from the first peak of the radial distribution function at c = 3 vol % (or wt %), where R_peak_ = 70 nm. The first peak of the structure factor should be located at *q*_*str*_ = 2π/*R*_*peak*_ ≤ 0.09 nm^–1^, which is very close to the lowest scattering wavevector accessible to our setup. Experiments on a wider *q*-range are envisaged to investigate the liquid-like structure crossing glass transition and the possible role played by micelle polydispersity in smearing out the structure peaks.^76^ This goes beyond the scope of the present work.

The effect of concentration on the radius of gyration, *R*_g_, of the micellar particles was studied through MD simulations. The latter was carried out for (i) micelles with fully charged PMAA arms, matching the experiments, and (ii) micelles with neutral PMAA arms as a comparison. Figure 3a depicts the change in the radius of gyration, *R*_g_ for the charged and neutral micelles with varying particle volume fractions. Importantly, the simulated volume fraction approximates the experimental mass fraction because each simulation bead has a mass of 1 (in simulation units) and the polymer density is assumed to be ∼ 1 g/cm^3^. A fully extended arm, similar to a rigid rod, would measure 183 nm in length. In the simulations, the average size of the arms, in the dilute regime, is ∼ 150 nm, suggesting that the arms are not fully stretched, a feature arising from local thermally induced bending along the arms’ backbone, in agreement with previous simulations of multiarm polyelectrolyte stars.^77,78^

**Figure 3:**
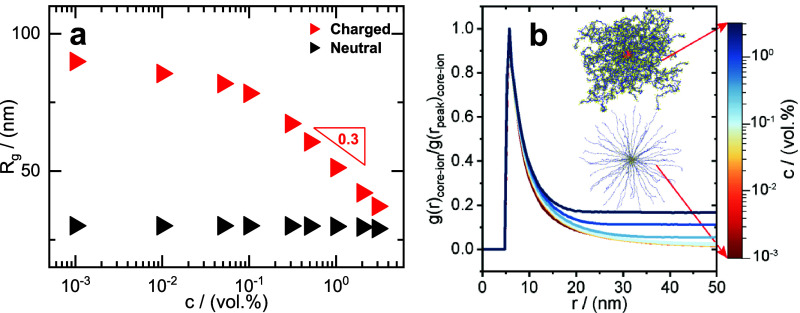
(a) Polymer concentration effect on the radius of gyration, *R*_g_, in both neutral and charged conditions obtained from MD simulations (see legend). (b) The normalized radial distribution function g­(*r*) between the micelle cores and the counterions in the solution in the MD simulations. The inset depicts simulation snapshots of the localization of the counterions around a micelle in the extremes of high and low concentration.

Simulations in the diluted regime showed that *R*_g_ starts at ∼ 90 nm (see also the Supporting Information for comparison with experiments) maintaining a faint plateau with an increase in concentration, and beyond a critical concentration of ∼ 0.25 vol % (or wt %), the *R*_g_ follows a power law of −0.3 reaching the value of 37 nm at the concentrated limit.

As will be seen later, this critical concentration corresponds well to the glass transition concentration observed in the linear rheology measurements (Figure 5). For the neutral counterpart (colloid with no charges), *R*_g_ remained constant with a value of ∼ 30 nm showing no dependence on polymer concentration. The lack of shrinkage for neutral colloids under the same conditions is a consequence of the fact that these are much smaller than their charged counterparts, placing their overlap concentration c* at a much higher value, so that, for a given value of monomer concentration *c*, the neutral particles are in the dilute regime but the polyelectrolyte micelles in the semidilute one.

To gain insight into the charge distribution of the micelles at different concentrations, the radial distribution of counterions has been inspected through simulations and reported in Figure 3b. The figure shows the radial distribution of counterions at distance *r* from the core of a single micellar polyelectrolyte particle g­(*r*)_core‑ion_ for varying concentrations and normalized by the value at the peak (the non-normalized plot is reported in Figure S1). In dilute conditions, most of the counterions are concentrated near the particle’s core. Few ions are present along the arms (moving away from the core) as evidenced by the sharper decay of the correlation function to zero. Hence, most parts of the arms have exposed charges that can interact with each other (interarm repulsion) through long-range unscreened electrostatic repulsions. The repulsion experienced between the arms leads to an extremely stretched configuration (as seen in the inset, Figure 3b). The electrostatic energy stemming from this uncompensated charge outweighs the energy associated with the fluctuating counterion gas within the micelle leading to strong repulsive interactions with neighboring (but far) particles giving rise to a correlated liquid.^56^ The net charge trapped in the bounding sphere of a micelle was also determined as a function of particle concentrations and plotted in Figure 4. Below the rheological glass transition (*c* < 0.25 wt %) the bounding sphere, enclosing a micelle, carries an effective negative charge, whereas in the glassy state, the effective charge is neutralized as a result of particle interdigitation.

As the polymer content increases (dark blue line in Figure 3b), the radial distribution function decays to a nonzero value because different micelles are now in contact and there is no space between them where the counterion density also decays to zero.

Figure 4 provides insights into the net charge distribution within the bounding sphere of a micelle as a function of concentration. In Figure 4a, the non-normalized net charge reveals that at low concentrations, the micelles retain an overall negative charge, indicating that not all counterions are bound to the polymer chains. This suggests that a significant fraction of counterions remains freely dispersed in the solvent. However, as concentration increases, especially across the rheological glass transition (concentration ∼ 0.25 vol %), as will be shown later, the net charge within the bounding sphere approaches zero, implying that a greater proportion of counterions become localized around the micelles, leading to effective charge screening and neutralization. This trend is a direct consequence of enhanced electrostatic interactions and confinement effects at higher concentrations.

**Figure 4:**
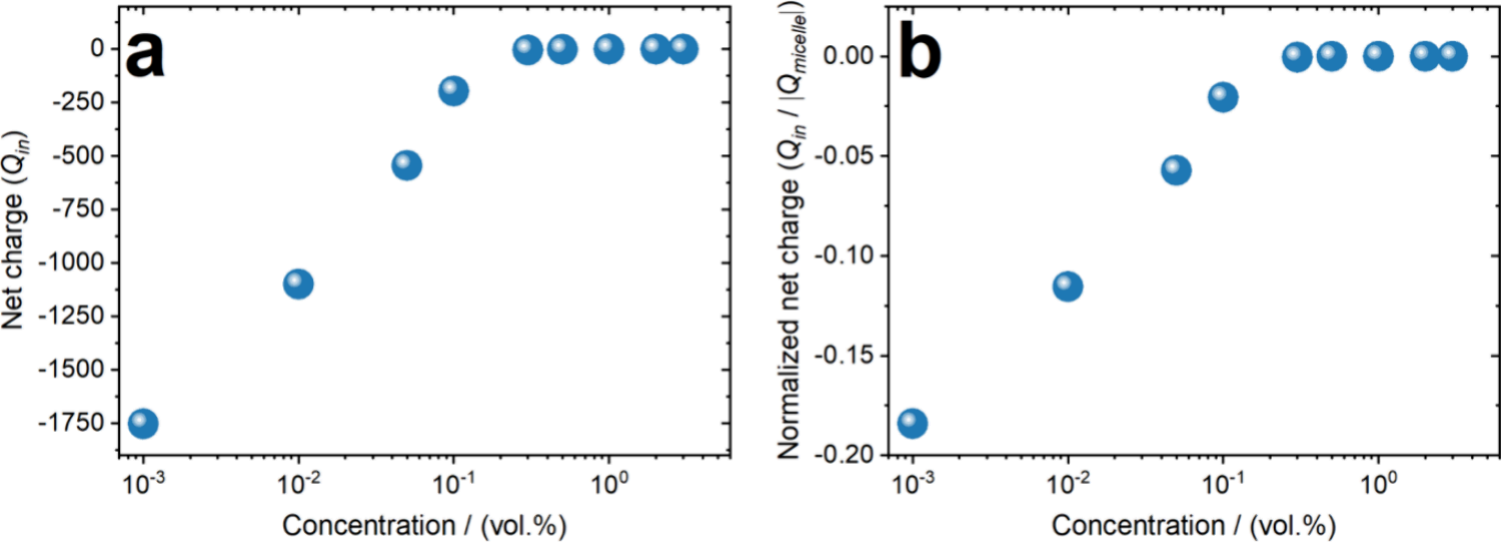
(a) Non-normalized and (b) normalized net charge trapped in the bounding sphere of a micelle as a function of varying concentrations obtained through MD simulations.

Figure 4b presents the normalized net charge, which accounts for variations in the total charge carried by the particle arms. The normalization highlights the progressive charge compensation occurring within the micellar environment as the system transitions toward higher concentrations. By ensuring that each counterion is assigned to the nearest micelle and avoiding double-counting, the analysis provides an accurate representation of the charge distribution. These results are critical for understanding the electrostatic interactions governing the behavior of charged micelles in solution, influencing their stability, aggregation, and overall dynamics. In particular, and in agreement with previous considerations pertaining to star-branched polyelectrolytes or brushes (see^79^), below the overlap concentration the interactions between the PE-stars are described by screened electrostatics whereas above c* the stars are overall neutral and thus the osmotic counterion pressure gives rise to a soft entropic repulsion between them.

### Glass Transition

Figure 5 shows the linear viscoelastic spectra of the multiarmed polyelectrolyte suspensions at various concentrations. At 0.1 and 0.2 wt %, the loss modulus (G”) is higher than the storage modulus (G’), indicating a viscoelastic liquid behavior. Unlike the low-concentration suspensions (below the glass transition) of neutral soft hairy colloids,^80^ which typically attain terminal flow, charged micelles do not show Newtonian behavior, even at mass fractions as low as 0.1 wt % (about 9c*). This is due to the significant arm stretching of the polyelectrolyte star polymers in the absence of salt.^45,52,74,81^ As a result, the fully stretched negatively charged arms cause long-ranged repulsive interactions among the particles, leading to the formation of a correlated liquid. Note that a neutral soft hairy colloidal system at 9c* would already exhibit a viscoelastic solid response due to the expected strong particle shell interdigitation with the nearest neighbors.

**Figure 5:**
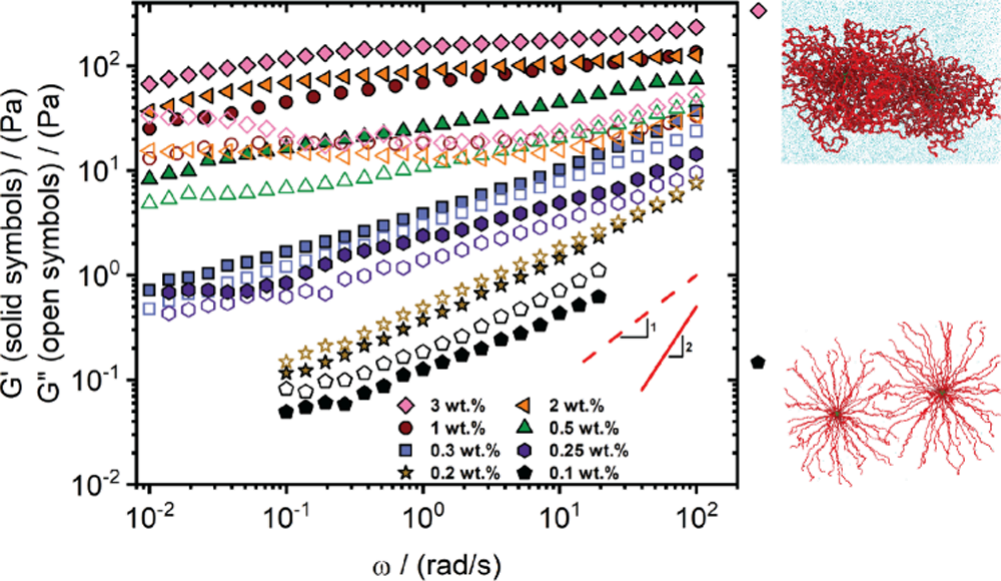
Linear viscoelastic spectra in terms of storage G’ (solid symbols) and loss G″ (open symbols) modulus as a function of the oscillation frequency ω for the multiarmed PS–PMAA polyelectrolyte solutions across varying concentrations (see legend). Experiments were carried out at 20 °C. The two snapshots from MD simulations depict the different behaviors of the multiarmed charged particles at varying concentrations. The snap at the bottom right corner (black symbol) represents the state of a correlated liquid corresponding to 0.1 wt %, and the snap at the top (pink symbol) shows the interdigitation of two particles deep in the glass state at 3 wt %, wherein the arms of the particles bend and engage in interpenetration. The complex viscosity dependence on angular frequency across the investigated concentrations is reported in Figure S4.

By contrast, we recall here, that suspensions of neutral soft hairy colloids exhibit a sharp glass transition as particle concentration increases:^80,82–84^ the formation of colloidal cages, where the outer hairy shells of particles interpenetrate, leads to an abrupt dynamic arrest of the suspensions.^14,80^ As a result, the elastic response largely outweighs the viscous contribution, leading to a frequency-independent response. Stress relaxation occurs either by in-cage particle rattling or arm disentanglement and hopping dynamics, depending on particle softness.^85^

Foffi et al.,^82^ combining statistical-mechanical theories and neutron scattering techniques on neutral star polymers, showed that the ratio between the number density at the glass transition and the overlap concentration is ∼ 1.4. This reflects strong shell interdigitation, which ultimately drives the dynamic arrest of the system. For the charged systems studied in this work, strong interdigitation occurs within the glass regime, beyond the concentration at which the system is dynamically arrested, and the particle arrangement is that of loose cages, as will be discussed further below. As a result, the ratio between the fraction at the glass transition (0.25 wt %) and the overlap concentration (0.01 wt %) is 25, a significantly higher value compared to neutral soft colloids. This comparison between the two systems needs to be accompanied by a qualification of two important differences between them: first, the charged micelles of this study shrink drastically until they reach their dynamical arrest. Indeed, based on Figure 3a, the particle shrinkage across the rheological glass transition (0.025 wt %) is ∼ 40%, resulting in an effective overlap concentration *c*_eff_* = 0.05 wt %. That is, at dynamical arrest (0.025 vol %), *c*/*c*_eff_* is approximately 5, yet quite larger compared to that expected for neutral soft colloids, ∼ 1.4. Second, in the case of charged micelles, the counterions play a very important role in determining the effective interactions between them^21,77–79,86^ and, thus, in bringing about liquid-like correlations and structural arrest in the system. We will return to this point later.

Indeed, raising the concentration of the charged micelles to 0.25 wt % results in a weak, frequency-dependent solid-like response that persists over four decades in frequency, and indicates a dynamically arrested state.^87–91^ As can be seen in Figure 4, at this concentration the counterions are all reabsorbed in the micelles’ interiors and thus the aggregates are overall neutral. Their effective interactions at this stage are dominated by the soft yet strong (≫ *k*_B_ *T*) entropic repulsions between the counterionic gas in their interiors^21,77–79^ with residual screened Coulombic repulsions between the increasingly overlapping arms, see Figure 7 and the discussion there. Accordingly, the system arrests by the formation of soft or loose cages, characteristic of glass formation for soft interactions. The latter concept has been recently posited by Sposini et al.,^92^ who studied the supercooled dynamics of the Gaussian core model. The authors established that the dynamics of the high particle density regime are not captured by the classic caging mechanism. Conversely, particles undergo a more continuous motion, better described by introducing the concept of loose cages. Additionally, this glass transition concentration corresponds well to the critical concentration at which there is a change in the correlation between *R*_g_ and particle concentration (Figure 3a).

At 0.3 wt %, (blue square symbols in Figure 5) the glassy state remains strongly frequency-dependent, implying weak interdigitation between neighboring particle shells. Increasing the mass concentration to 0.5 wt %, reduces the frequency dependence of the moduli, suggesting stronger connectivity between particle shells. At higher concentrations, namely 1, 2, and 3 wt %, the glassy systems exhibited nearly frequency-independent moduli responses, indicating that interdigitation occurs only at higher concentrations well within their vitrified state (ϕ_eff_ > 91). Systems well within the glass regime (>1 wt %) show a minimum in G”, with the corresponding inverse frequency indicating the time scale of the in-cage dynamics (β-relaxation),^14^ suggesting a transition from loose to tight cages. This transition is fully consistent with the fact that upon the increase of concentration the arms further collapse, promoting an inhomogeneous, semidilute polymer solution akin to that occurring for neutral stars. The resulting strong entropic repulsions between the chains, enhanced by the entropic repulsions of the trapped counterion clouds and residual Coulomb repulsions from closely approaching arms, bring forward a strengthening of the overall concentration-dependent effective repulsions between the micelles and thus a stiffening of the cages.

Shusharina and Rubinstein posited that for polyelectrolyte stars, increasing polymer concentration reduces the stars’ radii as the distance between their centers of mass decreases. This allows more stars to fit in a space-filling configuration before the onset of interdigitation.^52,59,93^ Consequently, the repulsive multiarmed polyelectrolyte particles change conformations within their glassy regime, with arms bending and slightly interpenetrating at higher concentrations, resulting in an almost frequency-independent response.

The linear viscoelastic data indicate that the vitrification of hairy charged systems, in the absence of salt, differs quite significantly from their neutral counterparts. In the dilute regime, the significantly stretched configuration of the outer particle shell drives the systems to a correlated liquid. As the concentration attains 0.25 wt %, an arrested state can be observed. A progressive increase of the particle concentration promotes a continuous change in the conformation of the particle’s arm, bending and compacting to accommodate more particles, leading to strong shell interpenetration way deep in the glassy regime, and exhibiting characteristics similar to neutral soft glasses.

Finer microscopic details of the rich phase behavior of the multiarmed polyelectrolyte particles observed in shear rheology are obtained through coarse-grained molecular dynamics simulations. At the molecular scale, a single-particle system comprises a glassy, hydrophobic PS core with PMAA serving as the arms in the corona. The simulations were performed with either (i) micelles with fully charged PMAA arms (same as the experiments) or (ii) micelles with neutral PMAA arms as a baseline comparison to traditional neutral colloids. Figure 6 depicts the radial distribution function g­(*r*)_core–core_ of the star polyelectrolytes going from diluted to a higher concentration of polyelectrolyte solution. As evident from Figure 6a, there are distinct peaks across all concentrations, ranging from the diluted to the concentrated regime. The appearance of these peaks is a clear indication of particle–particle correlation (like molecular liquid or colloid).^94^ Curiously, even at very low concentrations, such as 0.001 vol %, we observe a first-shell peak, suggesting that the stretched conformation of the corona and the long-range interactions organize the particles in loose colloidal cages. This result contrasts with the phenomenology encountered for neutral PMAA arms, where no pronounced peaks in g­(*r*)_core–core_ emerge at such low concentrations (Figure S2). This confirms that the charged micellar system exhibits long-range particle–particle interactions giving rise to a strongly correlated liquid at low volume concentrations, as concluded from Figure 5 at c = 0.1 wt %. Additionally, it was observed that with increasing concentration, there was a shift in the distribution function to lower values of *r* (particle core–core distance), with a power law dependence of the peak length scale R_peak_ on concentration, exhibiting a geometric scaling of 1/3 as reported in the inset of Figure 6a. The effect of concentration on g­(*r*)_core–core_ function is much smaller for micelles with neutral PMAA arms (see Figure S2). This effect is in agreement with earlier theoretical predictions based on effective interactions between charged star polymers,^86^ where it has been shown that for a fixed packing fraction, the resulting correlation functions in the presence of charges feature stronger liquid-like ordering than those for neutral stars. These stem from the simultaneous presence of Coulombic and counterion-entropic repulsions in the case of charged stars, as opposed to solely excluded-volume entropic repulsions for neutral star polymers.^82,95^

**Figure 6:**
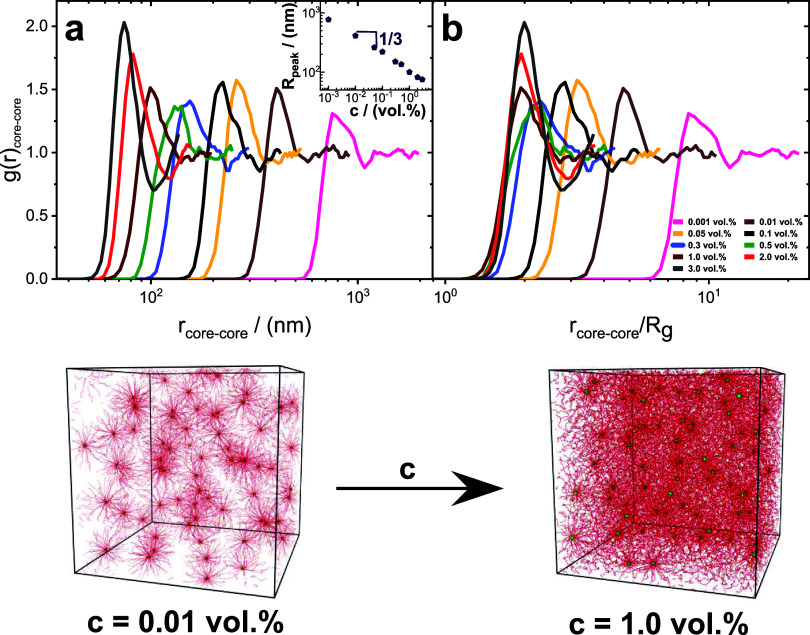
(a) The radial distribution function of the multiarmed PS–PMAA polyelectrolyte particles across varying concentrations (see legend). The inset represents the power law behavior between the average distance between the cores, R_peak,_ and concentration (with the geometric scaling of 1/3, as shown in the inset). (b) The radial distribution function of the multiarmed PS–PMAA polyelectrolyte particles rescaled with the radius of gyration, *R*_g_, at various concentrations (see legend). Snapshots from the MD simulations show the packing of the charged micellar particles upon increasing concentration.

In Figures 6a and 6b, the radial distribution function, g­(*r*), displays an interesting trend as the concentration (*c*) is increased. Initially, the height of the first peak of g­(*r*) increases, then decreases, and then rises again, with the minimum observed between 0.3 vol % and 0.5 vol %. This behavior reflects the melting process that soft colloids undergo, as they approach the jamming threshold.^11,96^ The fact that g­(*r*) continues to rise as the concentration increases beyond this threshold, represents a behavior that has been observed in other soft colloids.^97,98^

As shown in Figure 6b, the distance *r*_core–core_ between the cores was scaled with the radius of gyration of the micellar particle, *R*_g_. The rescaled g­(*r*)_core–core_ distribution shows that at concentrations higher than the rheological correlated liquid (0.1 vol %) R_peak_/*R*_g_ becomes affine to *R*_g_ because the micelles are in contact. At lower concentrations, the distance R_peak_ keeps growing proportionally to the polymer concentration and the scaling does not lead to a collapse of the g­(*r*)_core–core_ curves. Remarkably, the results indicate that R_peak_ always remains greater than *R*_g_, suggesting no strong shell interdigitation between micelles, consistent with the loose-cage dynamics (strong frequency-dependent response) detected in the rheological results, across the glass transition. As shown in Figure 3(a), the star shrinks as the concentration increases. Consequently, while the average separation between the stars decreases with c in absolute units (see Figure 6a), it remains approximately 2*R*_g_ when normalized by the star size, which serves as a density-dependent scale for interpreting the correlation functions.

Simulating the dynamics of the PS–PMAA micelle system used in the present study requires a large computational time. Hence, the dynamics of a scaled-down version of the micelles were studied. Figure S3 depicts the mean square displacement (MSD) for the PS–PMAA particles with neutral and charged arms. It is evident from Figure S3a that for neutral systems, the dynamics observed in the MSD of the cores do not strongly depend on concentration; the physics is the same and the long-time behavior remains diffusive. In contrast, for the charged systems (Figure S3b), the particles progressively slow down at very long times with increasing concentration, indicative of glassy dynamics with a clear transition from long-time diffusive dynamics to dynamically arrested states.

The combined findings of linear rheology and MD simulations suggest that our system is a glass at high concentrations (Figure 5) and a correlated liquid in the semidilute regime (Figures 5 and 6), with a transition point at around 0.1 wt % - 0.25 wt %. The emergence of such an arrested state is correlated with the associated phenomena of shrinking and interdigitation between neighboring stars. Notably, the repulsive PMAA arms prevent micelle interpenetration even at high concentrations (or effective volume fractions), as we show via a contact analysis of intermicelle monomers in the MD simulations. Indeed, Figure 7 displays the number of contacts between monomers of charged and neutral micelles as a function of particle concentration. A contact is counted if two monomers of different micelles are within a cutoff distance of 2.5 nm, (the cutoff distance of the interaction potential, see Materials and Methods for details). For soft multiarmed polyelectrolytes, from the diluted to the concentrated regime, the number of contacts between the micelles remains practically zero. This finding indicates that across their entire glass transition, the repulsive particles change their conformation contracting the arms inside the star to accommodate other particles while avoiding contact with each other as can be seen from the bottom snapshot in Figure 7. We observe that only at a very high concentration of 3 vol %, there is some interdigitation (with the number of contacts around ∼ 10), indicating that the system starts to engage in interpenetration only deep into the glassy state, supporting our findings in Figure 5.

**Figure 7:**
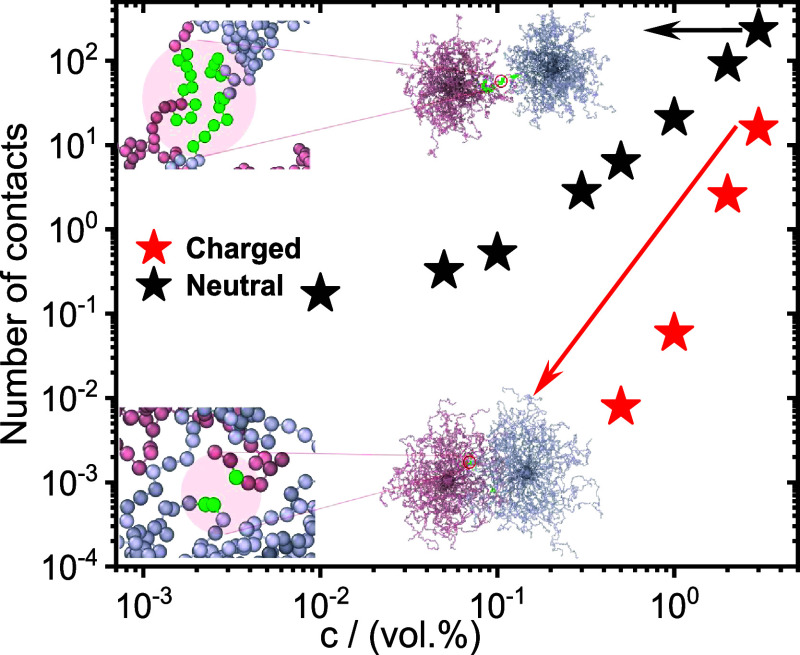
Interpenetration (contact monomers per micelle) between two simulated PS–PMAA polyelectrolyte particles as a function of concentration in charged and neutral conditions. Insets are snapshots from the MD simulations portraying the contact between the ends of the arms at the cutoff distance of 2.5 nm for charged and neutral systems.

Jusufi et al.^77,78^ performed computer simulations for two interacting polyelectrolyte stars and established that as their separation decreases beyond particle size, their arms retract to avoid interdigitation. The reason behind this behavior lies in the strong electric fields present in the stars’ interiors, whose strength scales with the distance *s* from the star center as ∼ 1/*s* so that insertion of the arms of the neighboring stars in that region would carry a very high electrostatic cost. In crowded solutions, when no space is available for the arms to escape, arm crumpling accompanied by (weak) interdigitation ensues. The same picture has emerged in the work of Wang and Denton,^98^ who studied the effective electrostatic interactions in solutions of polyelectrolyte stars with rigid arms and screened Coulomb interaction, highlighting the fact that interparticle interactions are highly influenced by arm anisotropy driven by electrostatic repulsion. As the stars approach each other, the forward arms are pushed back by interstar arm-arm interstar repulsion, but they can partially interdigitate at large concentrations due to rotational entropy. These insights support the idea that proximity and electrostatic repulsion influence configurational adjustments, aligning with our finding that charged micelles, in the absence of salt, tend to avoid overlap at low concentrations, with interdigitation appearing only under glassy conditions.

Conversely, for a neutral multiarmed particle, the arms can interpenetrate with each other, and at higher concentrations, the number of contacts reaches 150 as shown in the top snapshot of Figure 7. However, the value of 150 is still quite small compared to the number of beads per micelle, which amounts to 9000. This reflects the role of the unique architecture of the unimer and the resultant softer spherical micelles on their dynamics. Note the remarkable role played by the charged flexible arms of the particles that reduce contact with neighboring particle shells, despite getting closer upon increasing concentration.

### Nonlinear Viscoelasticity (NLVE)

The nonlinear viscoelastic behavior of the multiarmed PS–PMAA polyelectrolyte suspensions was assessed through start-up of shear experiments. Only the concentrated suspensions in the glass state were probed (see Figure 5). The time evolution of the shear stress σ^+^(*t*, γ̇) and the respective viscosity coefficient η^+^(*t*, γ̇) at various shear rates (γ̇) are reported in Figures S11 and S12, respectively.

In Figure 8a, the steady-state stress (σ_*ss*_) normalized by the thermal energy per unit particle volume *k*_*B*_ *T*/ ${R_{H}}^{3}$ is plotted as a function of the Peclet (*Pe*) number (eq 6), with the latter being the ratio of the advection, 1/γ̇, and diffusion, τ_*B*_ (eq 7), time.

$$Pe=\mathrm{\gamma ̇}\tau_{B}$$ 6

$$\tau_{B}=\frac{6\pi {R_{H}}^{3}\eta }{k_{B}T}$$ 7

where, *R*_*H*_ is the hydrodynamic radius, η is the viscosity of water, *k*_*B*_ is the Boltzmann constant, *T* is the temperature, and γ̇ is the shear rate.

**Figure 8:**
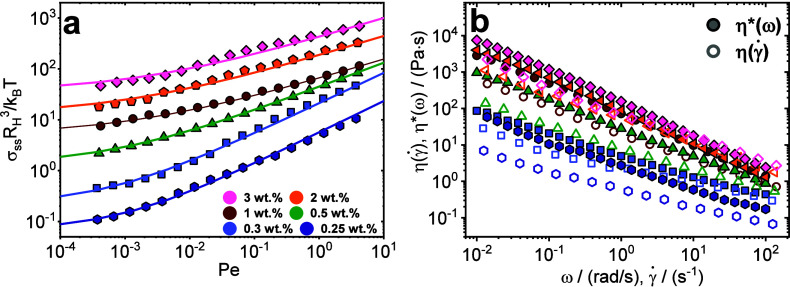
(a) Normalized flow curves as a function of Peclet number for multiarmed PS–PMAA polyelectrolyte solutions at varying concentrations (see legend). The lines are Herschel-Bulkley fits with parameters shown in Table 2. (b) Apparent steady-state and complex viscosities as a function of the shear rate and angular frequency, respectively, for diblock PS–PMAA polyelectrolyte solutions at varying concentrations (see legend).

The flow curves reported in Figure 8a extrapolate to a nonzero stress value at lower shear rates, indicating the presence of a yield stress, σ_*y*_.^99^ Such a response is typical of both hard and soft arrested systems.^8,17,100–103^ Consequently, the data fit fairly well with the Herschel-Bulkley equation,

$$\frac{\sigma_{ss}{R_{H}}^{3}}{k_{B}T}=\frac{\sigma_{y}{R_{H}}^{3}}{k_{B}T}+K{Pe}^{n}$$ 8

where, *K* is the consistency parameter and *n* is the flow index. The respective fitting parameters and the obtained yield stress values are reported in Table 2.

**Table 2:** Fitting Parameters for eq 8 for Varying Concentrations of Multiarmed Polyelectrolyte Solution: Concentration of the Solution, *c*; Effective Volume Fraction, ϕ_eff_; Yield Stress, σ_*y*_; Consistency, *K*; and Flow index, *n*

*c* **(wt %)**	**ϕ**_**eff**_	**σ**_*y*_ **(Pa)**	**σ**_*y*_ *R*_*H*_^**3**^/***k*_*B*_ *T***	***K***	***n***
3	273	22.9 ± 4.4	36.4	395 ± 6.3	0.39 ± 0.003
2	182	9.4 ± 2.2	12.9	175.7 ± 2.9	0.39 ± 0.006
1	91	3.3 ± 0.4	5.2	61.4 ± 0.4	0.39 ± 0.007
0.5	45	0.9 ± 0.2	1.4	43.4 ± 0.03	0.48 ± 0.001
0.3	27	0.13 ± 0.2	0.2	21 ± 0.2	0.60 ± 0.002
0.25	23	0.04 ± 0.01	0.07	5.6 ± 0.01	0.62 ± 0.006

The samples at all concentrations exhibit significant shear thinning behavior. However, at low *Pe* values (<0.00168), it is observed that the curves tend to a faint plateau and the dynamical yield stress of the material can be determined. The values of the yield stress corresponding to various concentrations are reported in Table 2. As expected, with increasing particle content, there is a rise in yield stress values: Larger stresses are needed to trigger material flow. Additionally, the flow index *n* has the highest value of 0.62 at the lowest concentration of 0.25 wt.% and proceeds to drop down to 0.39 upon entering further into their vitrified state. Index values between 0.71 – 0.32 ^19,104–108^ have been reported for varying colloidal glassy systems with a drop in the exponent value upon raising their packing fraction.^109–111^

In Figure 8b, the apparent steady-state and complex viscosities are plotted as a function of shear rate and angular frequency, respectively. This representation is usually employed to address the effect of shear on the structure of the system and, therefore, to test the so-called empirical Cox–Merz rule, according to which the nonlinear viscosity equals the linear complex viscosity if shear flows do not have a significant effect on the structure of the system. It is evident from Figure 8b that at all concentrations the complex viscosity is always higher than the viscosity measured under steady shear flow, as also observed in many other colloidal systems.^80,112–114^ As strong shear flow is imparted to the system, the colloidal cages are disrupted by the flow, a solid-to-liquid transition occurs, and as a result, the viscosity of the dispersion is lower compared to that in quiescent conditions (or linear viscoelastic regime).

Stress overshoots are typically observed in neutral hard^102,110,115–117^ and soft^17,110,118,119^ colloidal glasses, as a result of shear-induced cage breaking.^17,109,120–123^ As depicted in Figure S12, the soft-charged colloids investigated in this work do not exhibit overshoots in their transient shear response until well into their glassy regime. This nonlinear feature sets soft charged systems apart, reflecting the fact that across the glass transition, the system, akin to a Wigner glass, consists of loose caging, with weak or not at all particle interdigitation, and therefore no mechanical energy can be stored by the system prior to yielding. At higher concentrations (>0.5 wt.%), the arms have bent and contracted well into their core exhibiting a shear response analogous to other traditional colloidal systems.^17,99,109,110,118,124^

In start-up of shear experiments, the static yield stress (σ_*y*,*static*_) is the stress required for commencing the flow of a material, as opposed to the dynamic yield stress (see eq 8 and Table 2) being the minimum stress required to maintain the flow.^125^ Thus, the static yield stress depicts the yield value of the material after quiescent conditions, before disrupting its structure, marking the end of its elastic deformation.^126^ In Figure 9a, the static yield stress normalized by the thermal energy per unit particle volume is depicted as a function of the ratio between the effective and the glass volume fraction (ϕ_eff_/ϕ_glass_), at various *Pe* numbers, for all the glassy systems investigated. It is possible to observe that at *Pe* = 3·10^–4^, there exists a much stronger correlation between the normalized static yield stress and concentration compared to *Pe* = 3, implying that the concentration effect is stronger when the thermal motions govern the particle dynamics. Indeed, when *Pe* < 1, the Brownian motion of the particles dominates over the shear effects. At *Pe* > 1, the applied flow overtakes the Brownian motion, inducing a significant structural change to the system’s structure, that ultimately leads to a weaker rise in static yield stress values with particle concentration.

**Figure 9:**
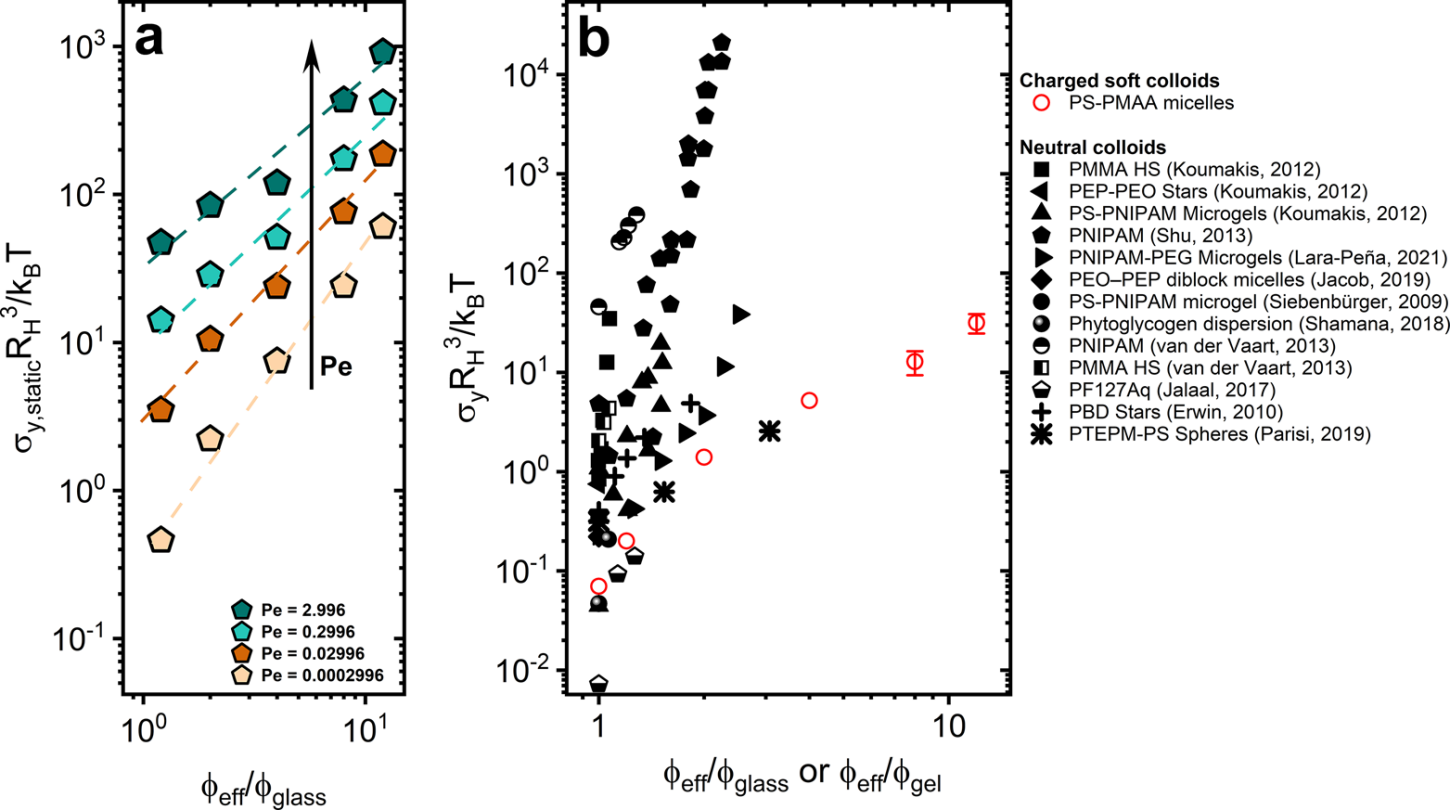
(a) Static yield stress normalized by the thermal energy per particle volume as a function of effective concentration across different Peclet numbers (see legend). The dashed lines are drawn as a guide to the eyes. (b) Yield stress normalized by the thermal energy per particle volume as a function of effective concentration, ϕ_eff_/ϕ_glass_ or ϕ_eff_/ϕ_gel_ for the multiarmed PS–PMAA polyelectrolyte solutions and other soft colloidal systems from literature (see legend).

Finally, to highlight the strong soft character exhibited by the multiarmed PS–PMAA polyelectrolyte stars used in this work, the normalized dynamic yield stress as a function of ϕ_eff_/ϕ_glass_ is reported in Figure 9b. In analogy with hard sphere system,^8^ the dynamic yield stress is normalized by *k*_B_ *T*/R_H_^3^, which is the expected magnitude of the yield stress at the glass transition and which contains the constant hydrodynamic radius *R*_H_, measured via dynamic light scattering in dilute conditions. As the particle concentration dependence of the colloidal shrinkage is difficult to assess, the particle length scale in dilute conditions is typically considered. Hence, the normalization factor *k*_B_ *T*/*R*_H_^3^ was utilized to study the softness among various systems as done previously in literature.

In the Figure 9b, a broad variety of colloidal glasses and gels from the literature^80,100,110,119,127–132^ are reported for comparison (see also the legend in Figure 9b). The normalization of the dynamic yield stress by the thermal energy per unit particle volume may not necessarily be strictly rigorous for all the reported systems, possibly due to attractive interactions between colloids, but it is adopted only for comparative purposes. Hard spheres (black square symbols) exhibit the strongest dependence on volume fraction, as particles cannot deform with increasing packing. Soft colloids include a variety of microgels, star polymers, and diblock copolymer micelles, which, depending on their softness, exhibit a strong or weak concentration dependence on the yield stress. Typically, softer systems display a much weaker concentration dependence due to their ability to deform to accommodate more particles in the same volume. Remarkably, in the case of multiarmed PS–PMAA polyelectrolyte particles (red circles), large volume fractions can be attained, with the yield stress depending very weakly on ϕ_eff_. With increasing concentration, these particles retract their negatively charged arms within their cores, preventing interpenetration, even for concentration well above the onset of the glassy behavior.

## Conclusions

The glass transition of a model system of salt-free, soft multiarm charged colloids has been assessed. The presence of charges significantly alters the glass transition compared to that observed in neutral colloids, with these differences identified through shear rheology, scattering techniques, and MD simulations.

In the liquid state, particles were strongly correlated via long-range repulsive interactions due to the negatively charged and significantly stretched outer shells. This resulted in a non-Newtonian behavior in shear rheology, characteristic of a correlated polymeric liquid. The particle core–core radial distribution function exhibited a strong correlation peak at volume fractions well below the overlap concentration, supporting the idea that charged systems interact strongly at distances larger than the individual particle size. This is not the case in neutral star polymers. As the particle volume fraction increased, dynamic arrest emerged, giving rise to a viscoelastic solid with strongly frequency-dependent moduli. This suggests weak interpenetration of particle shells, leading to the formation of loose colloidal cages that allow the system to be dynamically arrested but with a labile structure. A finer contact point analysis via MD simulations revealed that the outer shells exhibit few or no contact points until the system enters deep into the glassy regime. This unique behavior likely originates from the fact that the entropic and electrostatic costs for the shell–shell interdigitation outweigh the energy required for arm bending or retraction. However, overcrowding in the system inevitably led to arms interpenetration and particle size reduction due to space-filling constraints as confirmed by the particle form factors measured by SAXS.

Significant differences from neutral colloids were also observed in the transient shear measurements. The absence of shell–shell interdigitation across the glass transition and the consequent presence of loose colloidal cages render the investigated systems among the softest colloids reported in the literature. This was also reflected in the volume fraction dependence of the yield stress, which resulted in much weaker than that of most of the soft colloidal systems, such as star polymers and microgels.

The findings of this work provide significant and original insights into the glass transition of soft-charged and hairy colloids. Given the growing interest in charged systems for developing advanced materials and biomedical applications, these insights could play a critical role in guiding future research and practical applications.

## Supplementary Material



## Notes

Counterions distribution, radial distribution function for neutral micelles, mean square displacement of scaled-down systems; complex viscosity as a function of frequency and particle mass fraction, rejuvenation and aging experiments, start up of shear rate experiments; considerations on length scales in the system (PDF)

## References

[1] Pusey P. N., van Megen W. (1987). Observation of a Glass Transition in Suspensions of
Spherical Colloidal Particles. Phys. Rev. Lett..

[2] Bonn D., Tanaka H., Wegdam G., Kellay H., Meunier J. (1999). Aging of a
Colloidal “Wigner” Glass. Europhys.
Lett..

[3] Mason T. G., Weitz D. A. (1995). Linear Viscoelasticity of Colloidal
Hard Sphere Suspensions
near the Glass Transition. Phys. Rev. Lett..

[4] Lu P. J., Weitz D. A. (2013). Colloidal
Particles: Crystals, Glasses, and Gels. Annu.
Rev. Condens. Matter Phys..

[5] Pusey P. N., Van Megen W. (1986). Phase Behaviour
of Concentrated Suspensions of Nearly
Hard Colloidal Spheres. Nature.

[6] Pusey P. N. (2008). Colloidal
Glasses. J. Phys.: Condens. Matter.

[7] Pusey, P. N. ; Hansen, J. P. ; Levesque, D. ; Zinn-Justin, J. Liquids, Freezing and the Glass Transition. Les Houches Summer Schools of Theoretical Physics Session LI (1989). North-Holland: Amsterdam, 1991.

[8] Petekidis G., Vlassopoulos D., Pusey P. N. (2004). Yielding and Flow of Sheared Colloidal
Glasses. J. Phys. Condens. matter.

[9] Mason T. G., Bibette J., Weitz D. A. (1996). Yielding
and Flow of Monodisperse
Emulsions. J. Colloid Interface Sci..

[10] van
Ruymbeke E., Pamvouxoglou A., Vlassopoulos D., Petekidis G., Mountrichas G., Pispas S. (2010). Stable Responsive Diblock
Copolymer Micelles for Rheology Control. Soft
Matter.

[11] Philippe A.-M., Truzzolillo D., Galvan-Myoshi J., Dieudonné-George P., Trappe V., Berthier L., Cipelletti L. (2018). Glass Transition
of Soft Colloids. Phys. Rev. E.

[12] Stellbrink J., Lonetti B., Rother G., Willner L., Richter D. (2008). Shear Induced
Structures of Soft Colloids: Rheo-SANS Experiments on Kinetically
Frozen PEP–PEO Diblock Copolymer Micelles. J. Phys.: Condens. Matter.

[13] Helgeson M. E., Wagner N. J., Vlassopoulos D. (2007). Viscoelasticity
and Shear Melting
of Colloidal Star Polymer Glasses. J. Rheol.
(N. Y. N. Y)..

[14] Vlassopoulos D., Fytas G., Cloitre M. (2009). From Polymers to Colloids: Engineering
the Dynamic Properties of Hairy Particles. Adv.
Polym. Sci..

[15] Christopoulou C., Petekidis G., Erwin B., Cloitre M., Vlassopoulos D. (2009). Ageing and
Yield Behaviour in Model Soft Colloidal Glasses. Philos. Trans. R. Soc. A Math. Phys. Eng. Sci..

[16] Cloitre M., Borrega R., Leibler L. (2000). Rheological
Aging and Rejuvenation
in Microgel Pastes. Phys. Rev. Lett..

[17] Carrier V., Petekidis G. (2009). Nonlinear Rheology of Colloidal Glasses of Soft Thermosensitive
Microgel Particles. J. Rheol. (N. Y. N. Y)..

[18] Purnomo E.
H., van den Ende D., Vanapalli S. A., Mugele F. (2008). Glass Transition and
Aging in Dense Suspensions of Thermosensitive Microgel Particles. Phys. Rev. Lett..

[19] Cloitre M., Borrega R., Monti F., Leibler L. (2003). Glassy Dynamics and
Flow Properties of Soft Colloidal Pastes. Phys.
Rev. Lett..

[20] Mattsson J., Wyss H. M., Fernandez-Nieves A., Miyazaki K., Hu Z., Reichman D. R., Weitz D. A. (2009). Soft Colloids Make Strong Glasses. Nature.

[21] Jusufi A., Likos C. N. (2009). Colloquium: Star-Branched
Polyelectrolytes: The Physics
of Their Conformations and Interactions. Rev.
Mod. Phys..

[22] Wigner E. (1938). Effects of
the Electron Interaction on the Energy Levels of Electrons in Metals. Trans. Faraday Soc..

[23] Angelini R., Zaccarelli E., De Melo Marques F. A., Sztucki M., Fluerasu A., Ruocco G., Ruzicka B. (2014). Glass-Glass Transition during Aging
of a Colloidal Clay. Nat. Commun..

[24] Palberg T., Bartsch E., Beyer R., Hofmann M., Lorenz N., Marquis J., Niu R., Okubo T. (2016). To Make a Glass—Avoid
the Crystal. J. Stat. Mech. Theory Exp..

[25] Higler R., Krausser J., van der
Gucht J., Zaccone A., Sprakel J. (2018). Linking Slow
Dynamics and Microscopic Connectivity in Dense Suspensions of Charged
Colloids. Soft Matter.

[26] Cho H. W., Mugnai M. L., Kirkpatrick T. R., Thirumalai D. (2020). Fragile-to-Strong
Crossover, Growing Length Scales, and Dynamic Heterogeneity in Wigner
Glasses. Phys. Rev. E.

[27] Rosenberg R. O., Thirumalai D., Mountain R. D. (1989). Liquid, Crystalline and Glassy States
of Binary Charged Colloidal Suspensions. J.
Phys.: Condens. Matter.

[28] Beck C., Härtl W., Hempelmann R. (1999). The Glass
Transition of Charged and
Hard Sphere Silica Colloids. J. Chem. Phys..

[29] Abou B., Bonn D., Meunier J. (2001). Aging Dynamics
in a Colloidal Glass. Phys. Rev. E..

[30] Ruzicka B., Zulian L., Ruocco G. (2004). Ergodic to
Non-Ergodic Transition
in Low Concentration Laponite. J. Phys.: Condens.
Matter.

[31] Wu J., Liu Y., Chen W. R., Cao J., Chen S. H. (2004). Structural Arrest
Transitions in Fluids Described by Two Yukawa Potentials. Phys. Rev. E.

[32] Bandyopadhyay R., Liang D., Yardimci H., Sessoms D. A., Borthwick M. A., Mochrie S. G. J., Harden J. L., Leheny R. L. (2004). Evolution of Particle-Scale
Dynamics in an Aging Clay Suspension. Phys.
Rev. Lett..

[33] Lai S. K., Ma W. J., van Megen W., Snook I. K. (1997). Liquid-Glass Transition
Phase Diagram for Concentrated Charge-Stabilized Colloids. Phys. Rev. E.

[34] Ruzicka B., Zulian L., Ruocco G. (2004). Routes to Gelation in a Clay Suspension. Phys. Rev. Lett..

[35] Sciortino F., Mossa S., Zaccarelli E., Tartaglia P. (2004). Equilibrium
Cluster Phases and Low-Density Arrested Disordered States: The Role
of Short-Range Attraction and Long-Range Repulsion. Phys. Rev. Lett..

[36] Sciortino F., Tartaglia P. (2005). Glassy Colloidal Systems. Adv.
Phys..

[37] Porpora G., Rusciano F., Guida V., Greco F., Pastore R. (2021). Understanding
Charged Vesicle Suspensions as Wigner Glasses: Dynamical Aspects. J. Phys.: Condens. Matter.

[38] Lindsay H. M., Chaikin P. M. (1982). Elastic Properties
of Colloidal Crystals and Glasses. J. Chem.
Phys..

[39] Bosse J., Wilke S. D. (1998). Low-Density Ionic Glass. Phys.
Rev. Lett..

[40] Zaccarelli E., Andreev S., Sciortino F., Reichman D. R. (2008). Numerical Investigation
of Glassy Dynamics in Low-Density Systems. Phys.
Rev. Lett..

[41] Negi A. S., Osuji C. O. (2010). Time-Resolved Viscoelastic
Properties during Structural
Arrest and Aging of a Colloidal Glass. Phys.
Rev. E.

[42] Ruzicka B., Zulian L., Zaccarelli E., Angelini R., Sztucki M., Moussaïd A., Ruocco G. (2010). Competing Interactions in Arrested
States of Colloidal Clays. Phys. Rev. Lett..

[43] Joshi Y. M., Patel S., Suman K. (2024). Aqueous Laponite®
Dispersions
Are Attractive Gels, Not Repulsive Wigner Glasses: A Critical Commentarya). J. Rheol. (N. Y. N. Y)..

[44] Borisov O. V., Birshtein T. M., Zhulina E. B. (1991). Collapse of Grafted Polyelectrolyte
Layer. J. Phys. II.

[45] Borisov O. V. (1996). Conformations
of Star-Branched Polyelectrolytes. J. Phys.
II.

[46] Borisov O. V., Zhulina E. B. (1997). Structure of Weakly Charged Polyelectrolyte Brushes:
Monomer Density Profiles. J. Phys. II.

[47] Borisov O. V., Zhulina E. B. (1998). Effects of Ionic Strength and Charge Annealing in Star-Branched
Polyelectrolytes. Eur. Phys. J. B - Condens.
Matter Complex Syst..

[48] Klein
Wolterink J., Leermakers F. A. M., Fleer G. J., Koopal L. K., Zhulina E. B., Borisov O. V. (1999). Screening in Solutions of Star-Branched
Polyelectrolytes. Macromolecules.

[49] Borisov O.
V., Zhulina E. B. (2002). Effect
of Salt on Self-Assembly in Charged Block Copolymer
Micelles. Macromolecules.

[50] Borisov, O. V ; Zhulina, E. B. ; Leermakers, F. A. M. ; Ballauff, M. ; Müller, A. H. E. Conformations and Solution Properties of Star-Branched Polyelectrolytes. In Self Organized Nanostructures of Amphiphilic Block Copolymers I; Müller, A. H. E. , Borisov, O. , Eds.; Springer Berlin Heidelberg: Berlin, Heidelberg, 2011; pp 1–55. 10.1007/12_2010_104.

[51] Uhlík F., Košovan P., Limpouchová Z., Procházka K., Borisov O. V., Leermakers F. A. M. (2014). Modeling of Ionization and Conformations
of Starlike Weak Polyelectrolytes. Macromolecules.

[52] Shusharina N. P., Rubinstein M. (2008). Concentration
Regimes in Solutions of Polyelectrolyte
Stars. Macromolecules.

[53] Likos C. N., Blaak R., Wynveen A. (2008). Computer Simulations
of Polyelectrolyte
Stars and Brushes. J. Phys.: Condens. Matter.

[54] Jusufi A., Konieczny M., Likos C. N. (2012). Complexation of Charged Colloids
with Polyelectrolyte Stars. Zeitschrift für
Phys. Chemie.

[55] Blaak R., Likos C. N. (2012). Complexation and Overcharging of Polyelectrolyte Stars
and Charged Colloids. J. Phys.: Condens. Matter.

[56] Liénafa L., Oberdisse J., Mora S., Monge S., Robin J.-J. (2011). Rheology
and SANS on PET-b-PLAc-b-P­(DMAEMAq) Triblock Copolymers: Impact of
the PET and Polyelectrolyte Chain Length. Macromolecules.

[57] Moinard D., Borsali R., Taton D., Gnanou Y. (2005). Scattering and Viscosimetric
Behaviors of Four- and Six-Arm Star Polyelectrolyte Solutions. Macromolecules.

[58] Korobko A. V., Jesse W., Egelhaaf S. U., Lapp A., van der
Maarel J. R. C. (2004). Do Spherical Polyelectrolyte Brushes Interdigitate? *Phys*. Rev. Lett..

[59] Korobko A. V., Jesse W., Lapp A., Egelhaaf S. U., Van Der
Maarel J. R. C. (2005). Structure of Strongly Interacting Polyelectrolyte Diblock
Copolymer Micelles. J. Chem. Phys..

[60] Matějíček P., Podhájecká K., Humpolíčková J., Uhlík F., Jelínek K., Limpouchová Z., Procházka K., Špírková M. (2004). Polyelectrolyte
Behavior of Polystyrene-Block-Poly­(Methacrylic Acid) Micelles in Aqueous
Solutions at Low Ionic Strength. Macromolecules.

[61] Raffa P., Brandenburg P., Wever D. A. Z., Broekhuis A. A., Picchioni F. (2013). Polystyrene-Poly­(Sodium
Methacrylate) Amphiphilic Block
Copolymers by ATRP: Effect of Structure, PH, and Ionic Strength on
Rheology of Aqueous Solutions. Macromolecules.

[62] Mendes P.
R. D. S., Alicke A. A., Thompson R. L. (2014). Parallel-Plate Geometry Correction
for Transient Rheometric Experiments. Appl.
Rheol..

[63] Kremer K., Grest G. S. (1990). Dynamics of Entangled
Linear Polymer Melts: A Molecular-dynamics
Simulation. J. Chem. Phys..

[64] Hockney, R. W. ; Eastwood, J. W. Computer Simulation Using Particles; Adam Hilger, 1988.

[65] Kröger M. (2004). Simple Models
for Complex Nonequilibrium Fluids. Phys. Rep..

[66] Mendes E., Schädler V., Marques C. M., Lindner P., Wiesner U. (1997). Electrostatics
in the Self-Assembly of Macromolecular Surfactants. Europhys. Lett..

[67] Pedersen J. S., Gerstenberg M. C. (1996). Scattering Form Factor of Block Copolymer
Micelles. Macromolecules.

[68] Rubinstein, M , Colby, R. H. Polymer Physics; Oxford university press: New York, 2003.

[69] Daoud M., Cotton J. P. (1982). Star Shaped Polymers:
A Model for the Conformation
and Its Concentration Dependence. J. Phys. Paris.

[70] Raffa P., Stuart M. C. A., Broekhuis A. A., Picchioni F. (2014). The Effect
of Hydrophilic and Hydrophobic Block Length on the Rheology of Amphiphilic
Diblock Polystyrene-b-Poly­(Sodium Methacrylate) Copolymers Prepared
by ATRP. J. Colloid Interface Sci..

[71] Parisi D., Ruiz-Franco J., Ruan Y., Liu C. Y., Loppinet B., Zaccarelli E., Vlassopoulos D. (2020). Static and Dynamic Properties of
Block Copolymer Based Grafted Nanoparticles across the Non-Ergodicity
Transition. Phys. Fluids.

[72] Vlassopoulos D., Cloitre M. (2014). Tunable Rheology of Dense Soft Deformable Colloids. Curr. Opin. Colloid Interface Sci..

[73] Loppinet B., Fytas G., Vlassopoulos D., Likos C. N., Meier G., Liu G. J. (2005). Dynamics of Dense
Suspensions of Star-Like Micelles
with Responsive Fixed Cores. Macromol. Chem.
Phys..

[74] Heinrich M., Rawiso M., Zilliox J. G., Lesieur P., Simon J. P. (2001). Small-Angle
X-Ray Scattering from Salt-Free Solutions of Star-Branched Polyelectrolytes. Eur. Phys. J. E.

[75] Gineste S., Di Cola E., Amouroux B., Till U., Marty J.-D., Mingotaud A.-F., Mingotaud C., Violleau F., Berti D., Parigi G., Luchinat C., Balor S., Sztucki M., Lonetti B. (2018). Mechanistic
Insights into Polyion Complex Associations. Macromolecules.

[76] Genix A.-C., Oberdisse J. (2023). On the Absence
of Structure Factors in Concentrated
Colloidal Suspensions and Nanocomposites. Eur.
Phys. J. E.

[77] Jusufi A., Likos C. N., Löwen H. (2001). Conformations and Interactions of
Star-Branched Polyelectrolytes. Phys. Rev. Lett..

[78] Jusufi A., Likos C. N., Löwen H. (2002). Counterion-Induced
Entropic Interactions
in Solutions of Strongly Stretched, Osmotic Polyelectrolyte Stars. J. Chem. Phys..

[79] Jusufi A., Likos C. N., Ballauff M. (2004). Counterion
Distributions and Effective
Interactions of Spherical Polyelectrolyte Brushes. Colloid Polym. Sci..

[80] Erwin B. M., Cloitre M., Gauthier M., Vlassopoulos D. (2010). Dynamics and
Rheology of Colloidal Star Polymers. Soft Matter.

[81] Pincus P. (1991). Colloid Stabilization
with Grafted Polyelectrolytes. Macromolecules.

[82] Foffi G., Sciortino F., Tartaglia P., Zaccarelli E., Verso F. Lo, Reatto L., Dawson K. A., Likos C. N. (2003). Structural
Arrest in Dense Star-Polymer Solutions. Phys.
Rev. Lett..

[83] Seghrouchni R., Petekidis G., Vlassopoulos D., Fytas G., Semenov A. N., Roovers J., Fleischer G. (1998). Controlling
the Dynamics of Soft
Spheres: From Polymeric to Colloidal Behavior. Europhys. Lett..

[84] Laurati M., Stellbrink J., Lund R., Willner L., Richter D., Zaccarelli E. (2005). Starlike Micelles with Starlike Interactions:
A Quantitative
Evaluation of Structure Factors and Phase Diagram. Phys. Rev. Lett..

[85] Parisi D., Buenning E., Kalafatakis N., Gury L., Benicewicz B. C., Gauthier M., Cloitre M., Rubinstein M., Kumar S. K., Vlassopoulos D. (2021). Universal
Polymeric-to-Colloidal
Transition in Melts of Hairy Nanoparticles. ACS Nano.

[86] Hoffmann N., Likos C. N., Löwen H. (2004). Structure and Phase Behavior of Polyelectrolyte
Star Solutions. J. Chem. Phys..

[87] Parisi D., Camargo M., Makri K., Gauthier M., Likos C. N., Vlassopoulos D. (2021). Effect of
Softness on Glass Melting and Re-Entrant
Solidification in Mixtures of Soft and Hard Colloids. J. Chem. Phys..

[88] Parisi D., Truzzolillo D., Slim A. H., Dieudonné-George P., Narayanan S., Conrad J. C., Deepak V. D., Gauthier M., Vlassopoulos D. (2023). Gelation and Re-Entrance in Mixtures of Soft Colloids
and Linear Polymers of Equal Size. Macromolecules.

[89] Truzzolillo D., Marzi D., Marakis J., Capone B., Camargo M., Munam A., Moingeon F., Gauthier M., Likos C. N., Vlassopoulos D. (2013). Glassy States in Asymmetric Mixtures of Soft and Hard
Colloids. Phys. Rev. Lett..

[90] Truzzolillo D., Vlassopoulos D., Gauthier M., Munam A. (2013). Thermal Melting in
Depletion Gels of Hairy Nanoparticles. Soft
Matter.

[91] Truzzolillo D., Vlassopoulos D., Munam A., Gauthier M. (2014). Depletion Gels from
Dense Soft Colloids: Rheology and Thermoreversible Melting. J. Rheol. (N. Y. N. Y)..

[92] Sposini V., Likos C. N., Camargo M. (2023). Glassy Phases
of the Gaussian Core
Model. Soft Matter.

[93] Korobko A. V., Jesse W., Egelhaaf S. U., Lapp A., Van Der
Maarel J. R. C. (2004). Do Spherical Polyelectrolyte Brushes Interdigitate?. Phys. Rev. Lett..

[94] Hansen, J. P. ; McDonald, I. R. Theory of Simple Liquids: With Applications to Soft Matter; Elsevier Science, 2013.

[95] Likos C. N., Löwen H., Watzlawek M., Abbas B., Jucknischke O., Allgaier J., Richter D. (1998). Star Polymers Viewed as Ultrasoft
Colloidal Particles. Phys. Rev. Lett..

[96] Zhang Z., Xu N., Chen D. T. N., Yunker P., Alsayed A. M., Aptowicz K. B., Habdas P., Liu A. J., Nagel S. R., Yodh A. G. (2009). Thermal
Vestige of the Zero-Temperature Jamming Transition. Nature.

[97] Watzlawek M., Löwen H., Likos C. N. (1998). The Anomalous Structure Factor of
Dense Star Polymer Solutions. J. Phys.: Condens.
Matter.

[98] Wang H., Denton A. R. (2005). Effective Electrostatic Interactions in Solutions of
Polyelectrolyte Stars with Rigid Rodlike Arms. J. Chem. Phys..

[99] Pellet C., Cloitre M. (2016). The Glass and Jamming Transitions of Soft Polyelectrolyte
Microgel Suspensions. Soft Matter.

[100] Siebenbürger M., Fuchs M., Winter H., Ballauff M. (2009). Viscoelasticity
and Shear Flow of Concentrated, Noncrystallizing Colloidal Suspensions:
Comparison with Mode-Coupling Theory. J. Rheol.
(N. Y. N. Y)..

[101] Besseling R., Isa L., Ballesta P., Petekidis G., Cates M. E., Poon W. C. K. (2010). Shear
Banding and Flow-Concentration
Coupling in Colloidal Glasses. Phys. Rev. Lett..

[102] Koumakis N., Laurati M., Egelhaaf S. U., Brady J. F., Petekidis G. (2012). Yielding of
Hard-Sphere Glasses during Start-Up Shear. Phys.
Rev. Lett..

[103] Fuchs M., Ballauff M. (2005). Flow Curves of Dense Colloidal Dispersions:
Schematic Model Analysis of the Shear-Dependent Viscosity near the
Colloidal Glass Transition. J. Chem. Phys..

[104] Nordstrom K. N., Verneuil E., Arratia P. E., Basu A., Zhang Z., Yodh A. G., Gollub J. P., Durian D. J. (2010). Microfluidic
Rheology of Soft Colloids above and below Jamming. Phys. Rev. Lett..

[105] Bonn D., Denn M. M., Berthier L., Divoux T., Manneville S. (2017). Yield Stress Materials in Soft Condensed
Matter. Rev. Mod. Phys..

[106] Basu A., Xu Y., Still T., Arratia P. E., Zhang Z., Nordstrom K. N., Rieser J. M., Gollub J. P., Durian D. J., Yodh A. G. (2014). Rheology
of Soft Colloids across
the Onset of Rigidity: Scaling Behavior­{,} Thermal­{,} and Non-Thermal
Responses. Soft Matter.

[107] Dhont J. K. G., Kang K., Kriegs H., Danko O., Marakis J., Vlassopoulos D. (2017). Nonuniform
Flow in Soft Glasses of
Colloidal Rods. Phys. Rev. Fluids.

[108] Bonnecaze, R. T. ; Cloitre, M. Micromechanics of Soft Particle Glasses. In High Solid Dispersions; Cloitre, M. , Ed.; Springer Berlin Heidelberg: Berlin, Heidelberg, 2010; pp 117–161. 10.1007/12_2010_90.

[109] Koumakis N., Petekidis G. (2011). Two Step Yielding
in Attractive Colloids:
Transition from Gels to Attractive Glasses. Soft Matter.

[110] Koumakis N., Pamvouxoglou A., Poulos A. S., Petekidis G. (2012). Direct Comparison
of the Rheology of Model Hard and Soft Particle Glasses. Soft Matter.

[111] Khabaz F., Cloitre M., Bonnecaze R. T. (2020). Particle
Dynamics Predicts Shear Rheology of Soft Particle Glasses. J. Rheol. (N. Y. N. Y)..

[112] Bertsch P., Sánchez-Ferrer A., Bagnani M., Isabettini S., Kohlbrecher J., Mezzenga R., Fischer P. (2019). Ion-Induced
Formation of Nanocrystalline Cellulose Colloidal Glasses Containing
Nematic Domains. Langmuir.

[113] Nzé R.-P., Nicolai T., Chassenieux C., Nicol E., Boye S., Lederer A. (2015). Effect of Connectivity
on the Structure and the Liquid–Solid Transition of Dense Suspensions
of Soft Colloids. Macromolecules.

[114] Siebenbürger M., Fuchs M., Ballauff M. (2012). Core–Shell Microgels
as Model Colloids for Rheological Studies. Soft
Matter.

[115] Jacob A. R., Moghimi E., Petekidis G. (2019). Rheological
Signatures of Aging in Hard Sphere Colloidal Glasses. Phys. Fluids.

[116] Laurati M., Mutch K. J., Koumakis N., Zausch J., Amann C. P., Schofield A. B., Petekidis G., Brady J. F., Horbach J., Fuchs M., Egelhaaf S. U. (2012). Transient
Dynamics in Dense Colloidal Suspensions under Shear: Shear Rate Dependence. J. Phys.: Condens. Matter.

[117] Sentjabrskaja T., Hermes M., Poon W. C. K., Estrada C. D., Castañeda-Priego R., Egelhaaf S. U., Laurati M. (2014). Transient
Dynamics during Stress Overshoots in Binary Colloidal Glasses. Soft Matter.

[118] Rogers S. A., Callaghan P. T., Petekidis G., Vlassopoulos D. (2010). Time-Dependent Rheology of Colloidal
Star Glasses. J. Rheol. (N. Y. N. Y)..

[119] Lara-Peña M. A., Licea-Claverie A., Zapata-González I., Laurati M. (2021). Colloidal
and Polymeric Contributions to the Yielding
of Dense Microgel Suspensions. J. Colloid Interface
Sci..

[120] Merola M. C., Parisi D., Truzzolillo D., Vlassopoulos D., Deepak V. D., Gauthier M. (2018). Asymmetric Soft-Hard
Colloidal Mixtures: Osmotic Effects, Glassy States and Rheology. J. Rheol. (N. Y. N. Y)..

[121] Koumakis N., Ballesta P., Besseling R., Poon W. C. K., Brady J. F., Petekidis G. (2013). Colloidal
Gels under Shear: Strain Rate Effects. AIP Conf.
Proc..

[122] Koumakis N., Laurati M., Jacob A. R., Mutch K. J., Abdellali A., Schofield A. B., Egelhaaf S. U., Brady J. F., Petekidis G. (2016). Start-up Shear
of Concentrated Colloidal Hard Spheres:
Stresses, Dynamics, and Structure. J. Rheol.
(N. Y. N. Y)..

[123] Sentjabrskaja T., Hendricks J., Jacob A. R., Petekidis G., Egelhaaf S. U., Laurati M. (2018). Binary Colloidal Glasses under Transient
Stress- and Strain-Controlled Shear. J. Rheol.
(N. Y. N. Y)..

[124] Derec C., Ducouret G., Ajdari A., Lequeux F. (2003). Aging and
Nonlinear Rheology in Suspensions of Polyethylene Oxide--Protected
Silica Particles. Phys. Rev. E.

[125] Dinkgreve M., Paredes J., Denn M. M., Bonn D. (2016). On Different
Ways of Measuring “the” Yield Stress. J. Nonnewton. Fluid Mech..

[126] De Kee D. (2021). Yield Stress Measurement Techniques:
A Review. Phys. Fluids.

[127] Shu R., Sun W., Liu Y., Wang T., Wang C., Liu X., Tong Z. (2013). The Jamming
and Unjamming Transition in Poly­(N-Isopropylacrylamide)
Microgel Suspensions. Colloids Surfaces A Physicochem.
Eng. Asp..

[128] Jacob A. R., Poulos A. S., Semenov A. N., Vermant J., Petekidis G. (2019). Flow Dynamics
of Concentrated Starlike Micelles: A
Superposition Rheometry Investigation into Relaxation Mechanisms. J. Rheol. (N. Y. N. Y)..

[129] Shamana H., Grossutti M., Papp-Szabo E., Miki C., Dutcher J. R. (2018). Unusual Polysaccharide
Rheology of
Aqueous Dispersions of Soft Phytoglycogen Nanoparticles. Soft Matter.

[130] van der Vaart K., Rahmani Y., Zargar R., Hu Z., Bonn D., Schall P. (2013). Rheology of Concentrated Soft and
Hard-Sphere Suspensions. J. Rheol. (N. Y. N.
Y)..

[131] Jalaal M., Cottrell G., Balmforth N., Stoeber B. (2017). On the Rheology of
Pluronic F127 Aqueous Solutions. J. Rheol. (N.
Y. N. Y)..

[132] Parisi, D. Tailoring the Rheology of Suspensions and Composites by Altering Shape and Compositions; University of Crete, 2019.

