# Supplementary material for: Glass Transition and Yielding of Ultrasoft Charged Spherical Micelles
Source: Macromolecules. 2025 Apr 30;58(18):9724–39. doi: 10.1021/acs.macromol.4c03215 (PMC12461935; doi:10.1021/acs.macromol.4c03215)
Supplement: Supplementary file 1 [file ma4c03215_si_001.pdf]

Supporting Information
Glass transition and yielding of ultrasoft charged spherical micelles

Roshan Akdar Mohamed Yunus[1], Utku Gürel[2], Aleksander Guzik[1], Philippe Dieudonne-George[3], Marc C.A. Stuart[4], Patrizio Raffa[1], Christos N. Likos[5], Domenico Truzzolillo[3], Andrea Giuntoli[2], and Daniele Parisi[1]

[1]*Department of Chemical Engineering, Engineering and Technology Institute Groningen, University of Groningen, Nijenborgh 3, 9747 AG Groningen, The Netherlands*
[2]*Zernike Institute for Advanced Materials Micromechanics, University of Groningen, Nijenborgh 3, 9747 AG Groningen, the Netherlands*
[3]*Laboratoire Charles Coulomb (L2C), UMR 5221 CNRS Université de Montpellier, Montpellier 34095, France*
[4]*Electron Microscopy, Groningen Biomolecular Sciences and Biotechnology Institute, University of Groningen, Nijenborgh 7, 9747 AG Groningen, the Netherlands*
[5]*Faculty of Physics, University of Vienna, Boltzmanngasse 5, 1090 Vienna, Austria*

Table of Contents

Molecular Dynamics (MD) Simulation **2**
Linear Viscoelasticity **4**
Rejuvenation and Aging **5**
Non-linear viscoelasticity **9**

Molecular Dynamics (MD) Simulation

The counterion distribution around a single PS-PMAA micelle is investigated through MD simulations and reported in Figure S1.

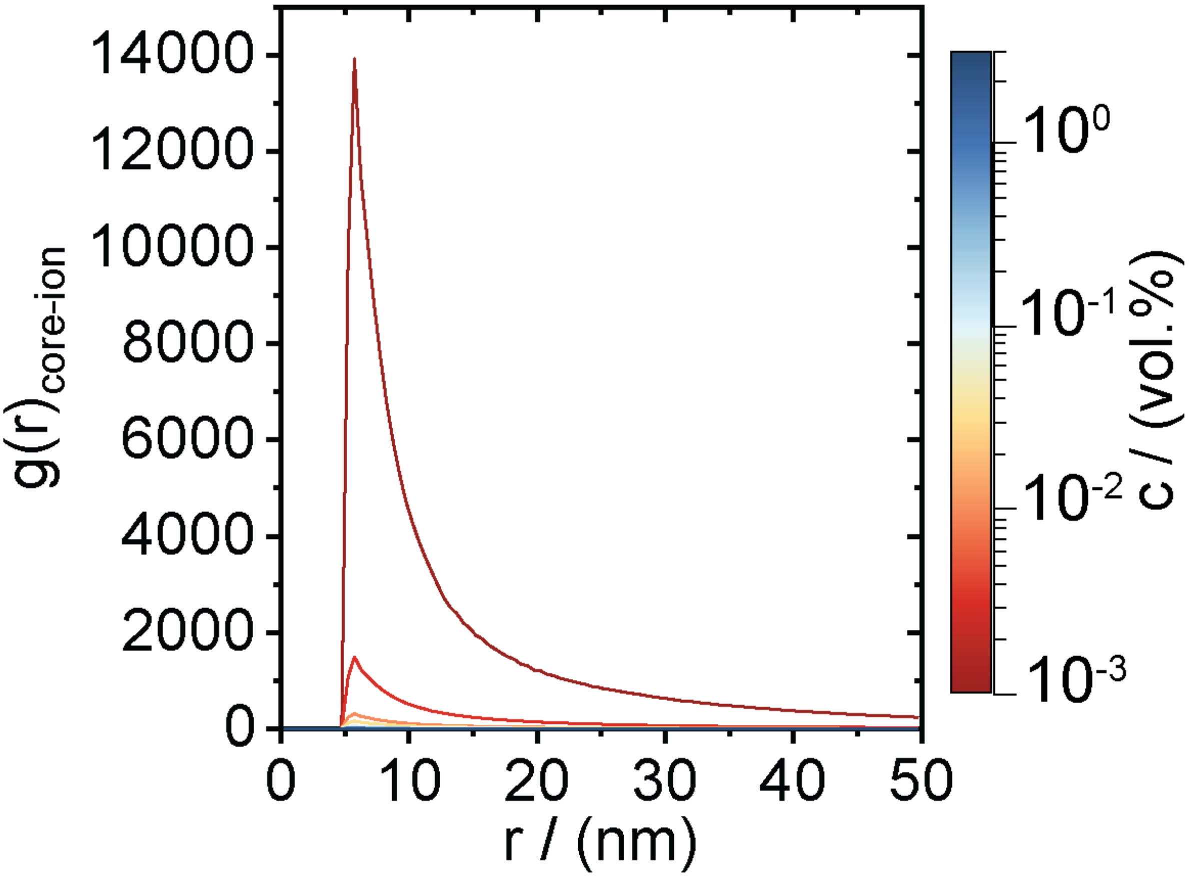

Figure S1. Radial distribution function g(*r*) between the micelle cores and the counterions in the solution in the MD simulations across varying concentrations.

Figure S2 depicts the radial distribution function of the multiarmed PS-PMAA neutral particles across different concentrations. At lower concentrations (< 0.5 vol.%), the distribution does not exhibit any distinct peaks unlike the charged particles (Figure 6) implying absence of strong correlation. Additionally, unlike the charged particles, the effect of concentration is much smaller on the radial distribution function.

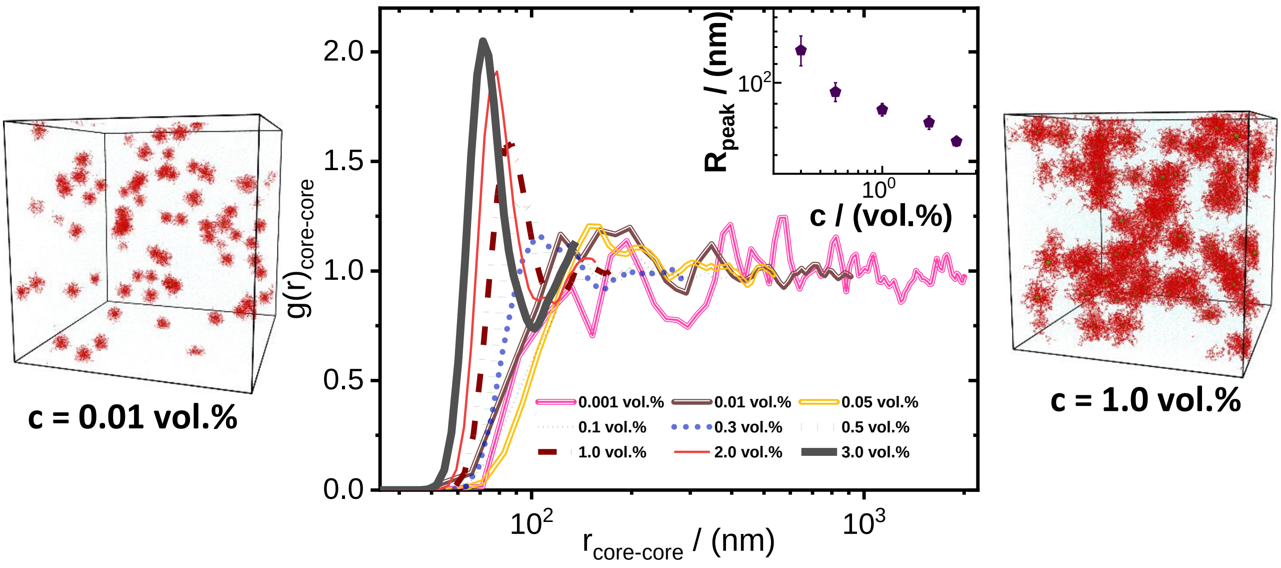

Figure S2. Radial distribution function of the multiarmed PS-PMAA neutral particles across varying concentrations (see legend). The inset represents the power law behavior between the average distance between the cores, R_{peak} and concentration. Snapshots from the MD simulations show the packing of the neutral micellar particles at dilute and concentrated regime.

Since the PS-PMAA system employed in the study is too big to extract the dynamics through MD simulations, studies were performed over smaller systems (with smaller cores, and shorter arms). Figure S3 shows the mean square displacement (MSD) for PS-PMAA particles with both neutral and charged arms. As seen in Figure S3a, the MSD of the cores in neutral systems remains unaffected by concentration since the particles do not interact. However, in charged systems (Figure S3b), the particles exhibit a gradual slowdown over extended timescales as concentration increases, suggesting more pronounced caging dynamics.

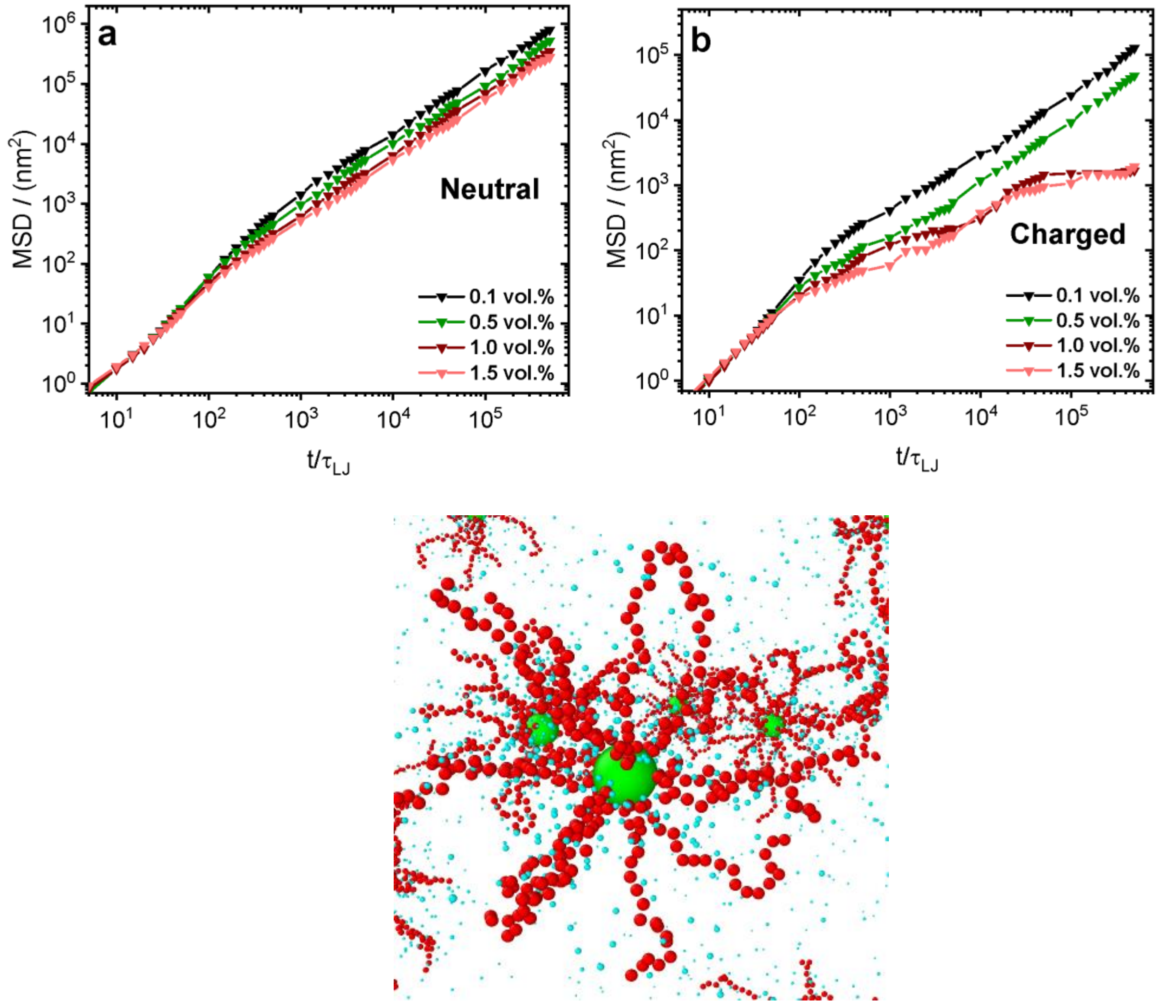

Figure S3. Mean square displacement (MSD) of the cores for a scaled down multiarmed PS-PMAA system (smaller core, shorter arms, as shown in snapshot) as a function of normalized time for neutral (a) and charged conditions (b) across varying concentration (see legend).

Linear Viscoelasticity

The linear viscoelastic properties of the multiarmed PS-PMAA polyelectrolyte solution were investigated through small amplitude oscillatory shear (SAOS) experiments. Figure S4 shows the complex viscosity as a function of angular frequency across varying concentrations. As seen from Figure S4, starting from the dilute concentration 0.1 wt.%, the material does not exhibit Newtonian behavior, rather shear thinning indicative of the strong correlation between the particles (as also evident from Figure 5). The correlation between complex viscosity and frequency gets stronger across glass transition (at 0.25 wt.%) and gets further strengthened as we move to higher concentrations (3 wt.%).

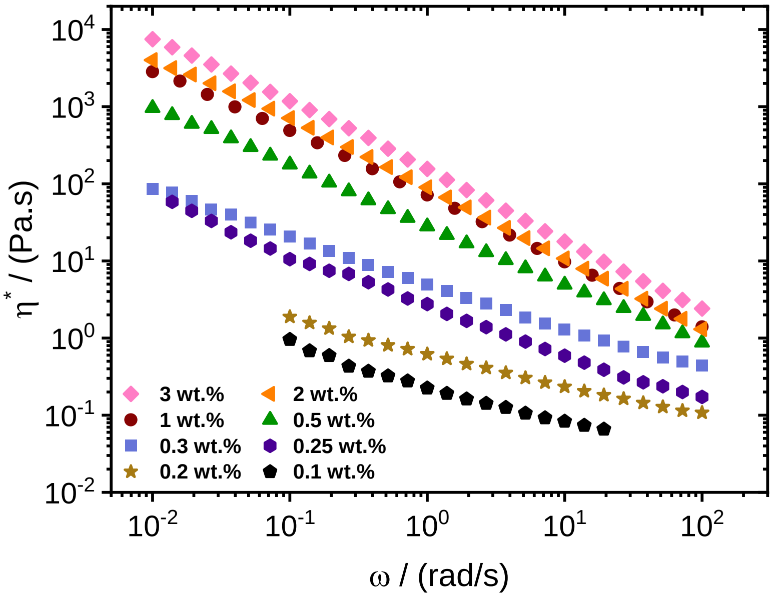

Figure S4. Complex viscosity η* as a function of angular frequency ω for the multiarmed PS-PMAA polyelectrolyte solution across varying concentrations (see legend).

Rejuvenation and Aging

As part of the experimental protocol, the material was subjected to rejuvenation and aging prior to any rheological measurement (both linear and non-linear shear rheology). The rejuvenation is a dynamic time sweep (DtS) at 1 rad/s and a selected 200% strain (ensured to choose a strain falling in the non-linear regime by performing a DSS at 1 rad/s), typically for 60 s – 100 s (until steady state was observed) to destroy the mechanical history of the sample. Subsequently, the aging is a DtS conducted at 1 rad/s and strain falling in LVE regime for 200 s to reconstruct the original structure of the material. Figures S5 to S10 depict the results of rejuvenation and aging for the charged multiarmed PS-PMAA solution across varying concentrations.

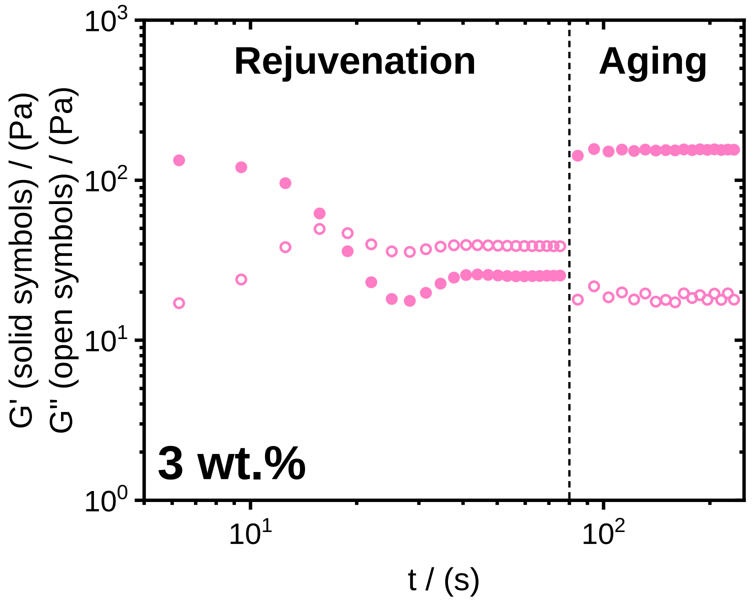

Figure S5. Dynamic time sweep (DtS) at 1 rad/s for 3 wt.% 4 multiarmed PS-PMAA polyelectrolyte solution. It depicts the structural breakdown of the sample at 200% strain and instant material recovery with strain in linear regime.

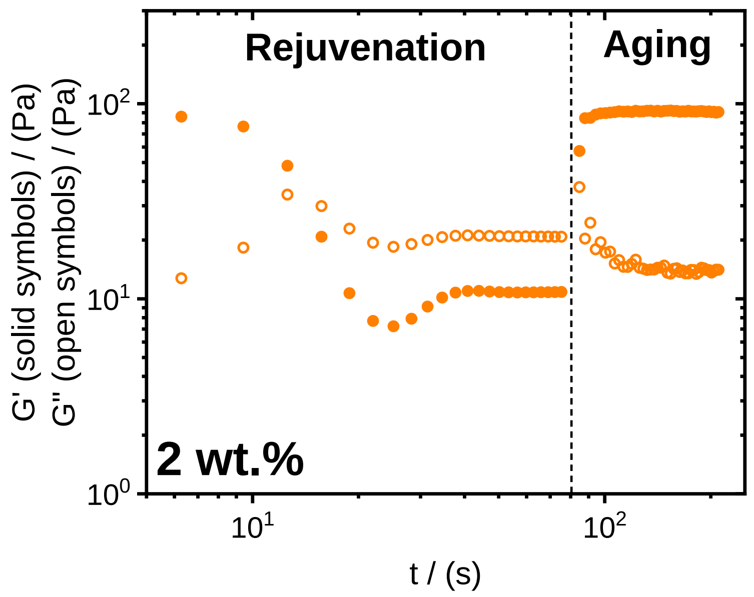

Figure S6. Dynamic time sweep (DtS) at 1 rad/s for 2 wt.% multiarmed PS-PMAA polyelectrolyte solution. It depicts the structural breakdown of the sample at 200% strain and instant material recovery with strain in linear regime.

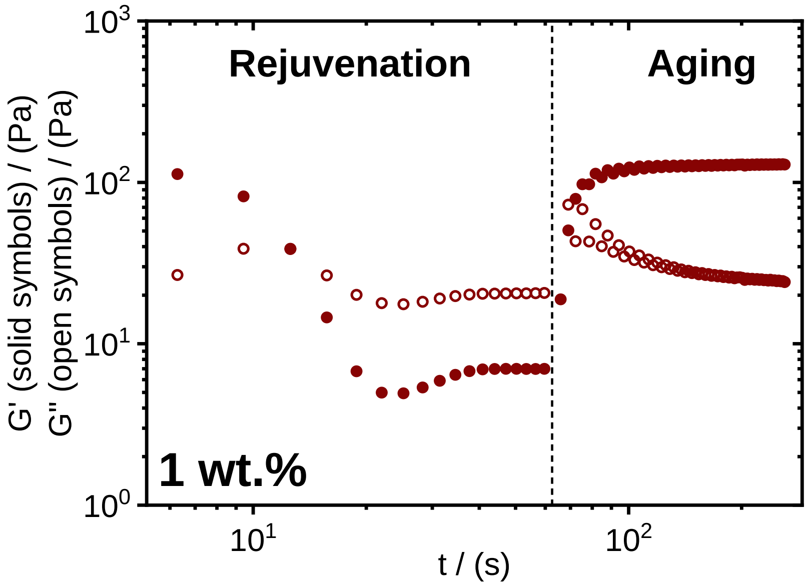

Figure S7. Dynamic time sweep (DtS) at 1 rad/s for 1 wt.% multiarmed PS-PMAA polyelectrolyte solution. It depicts the structural breakdown of the sample at 200% strain and instant material recovery with strain in linear regime.

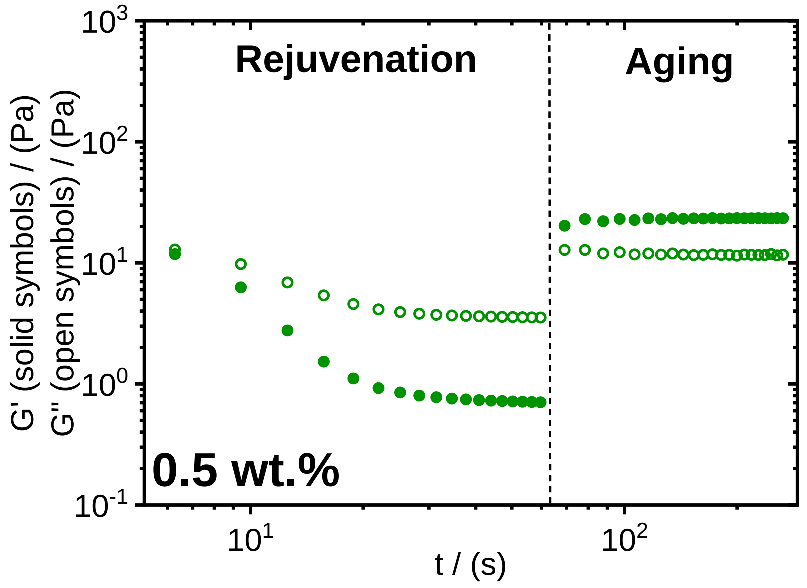

Figure S8. Dynamic time sweep (DtS) at 1 rad/s for 0.5 wt.% multiarmed PS-PMAA polyelectrolyte solution. It depicts the structural breakdown of the sample at 200% strain and instant material recovery with strain in linear regime.

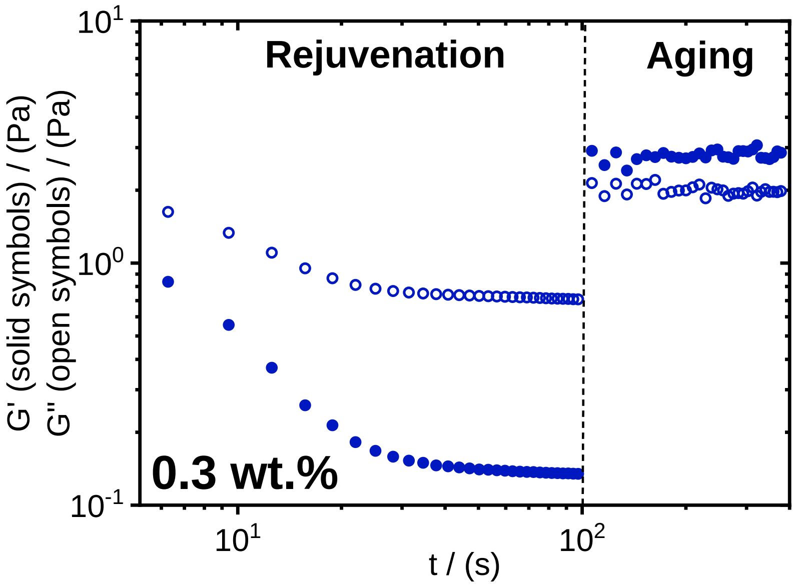

Figure S9. Dynamic time sweep (DtS) at 1 rad/s for 0.3 wt.% multiarmed PS-PMAA polyelectrolyte solution. It depicts the structural breakdown of the sample at 200% strain and instant material recovery with strain in linear regime.

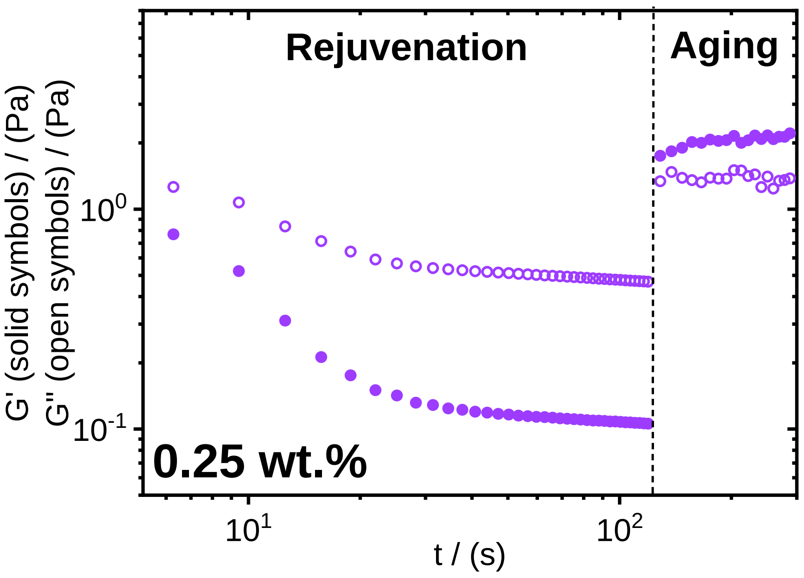

Figure S10. Dynamic time sweep (DtS) at 1 rad/s for 0.25 wt.% multiarmed PS-PMAA polyelectrolyte solution. It depicts the structural breakdown of the sample at 200% strain and instant material recovery with strain in linear regime.

Non-linear viscoelasticity

The non-linearity of multiarmed PS-PMAA polyelectrolyte solution across different concentrations were investigated through start-up of shear experiments, reported in Figures S11 - S12.

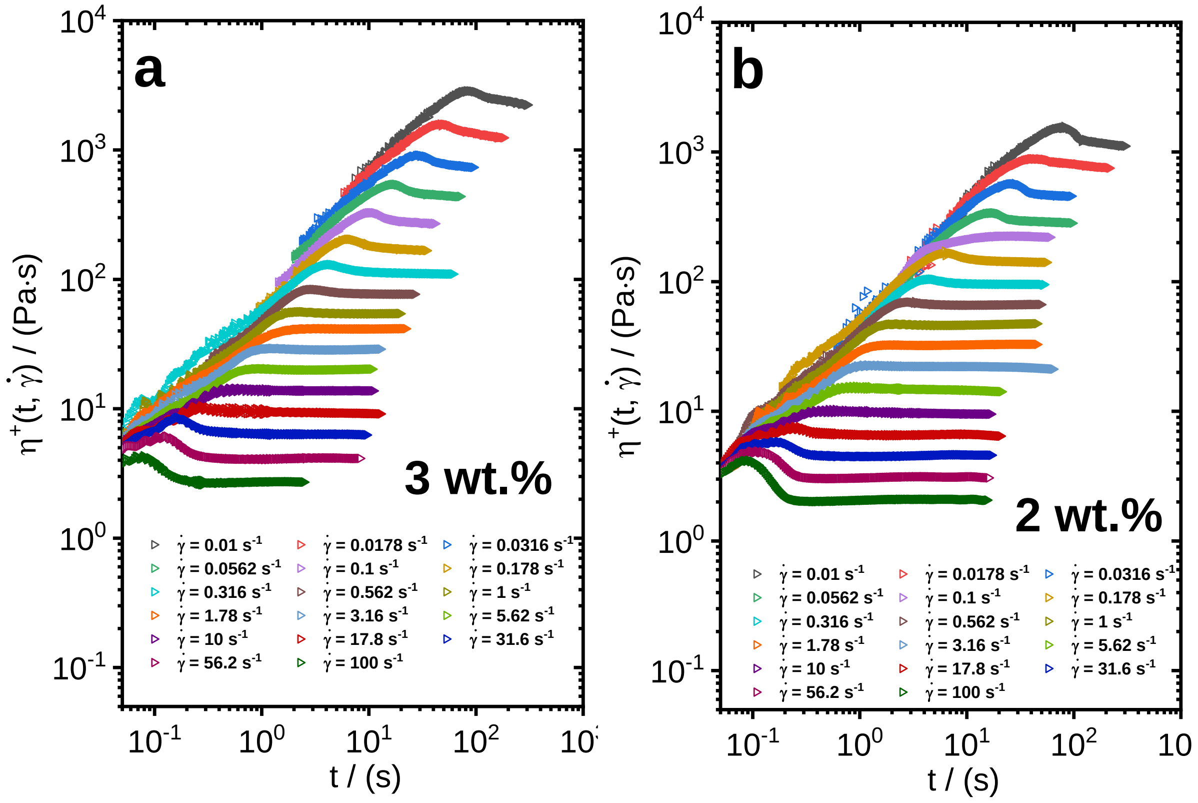

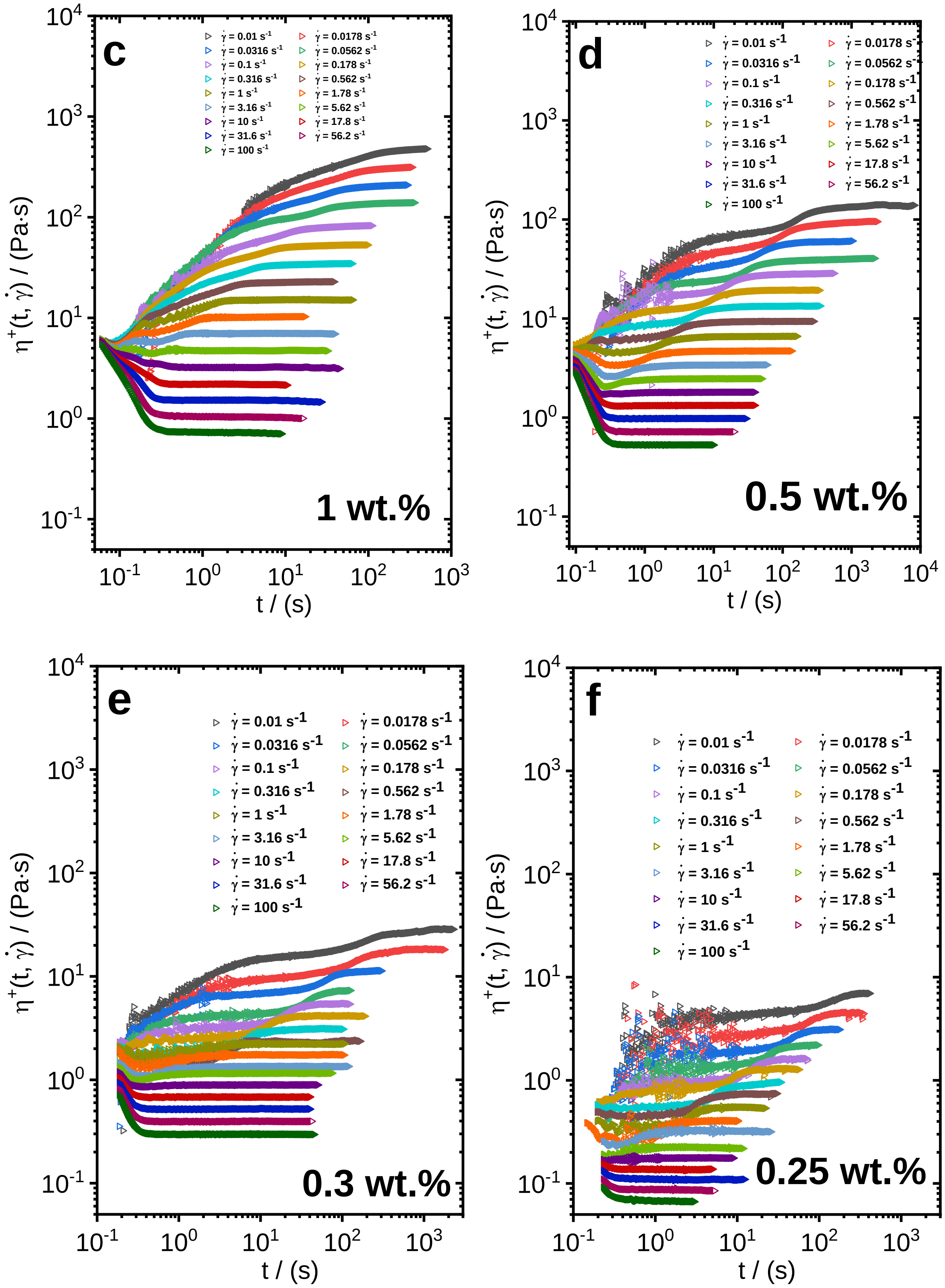

Figure S11. Start-up of the shear rate in terms of the stress growth coefficient as a function of time at various shear rates (reported in the legend) for multiarmed PS-PMAA polyelectrolyte solution at (a) 3 wt.%, (b) 2 wt.%, (c) 1 wt.%, (d) 0.5 wt.%, (e) 0.3 wt.%, and (f) 0.25 wt.%.

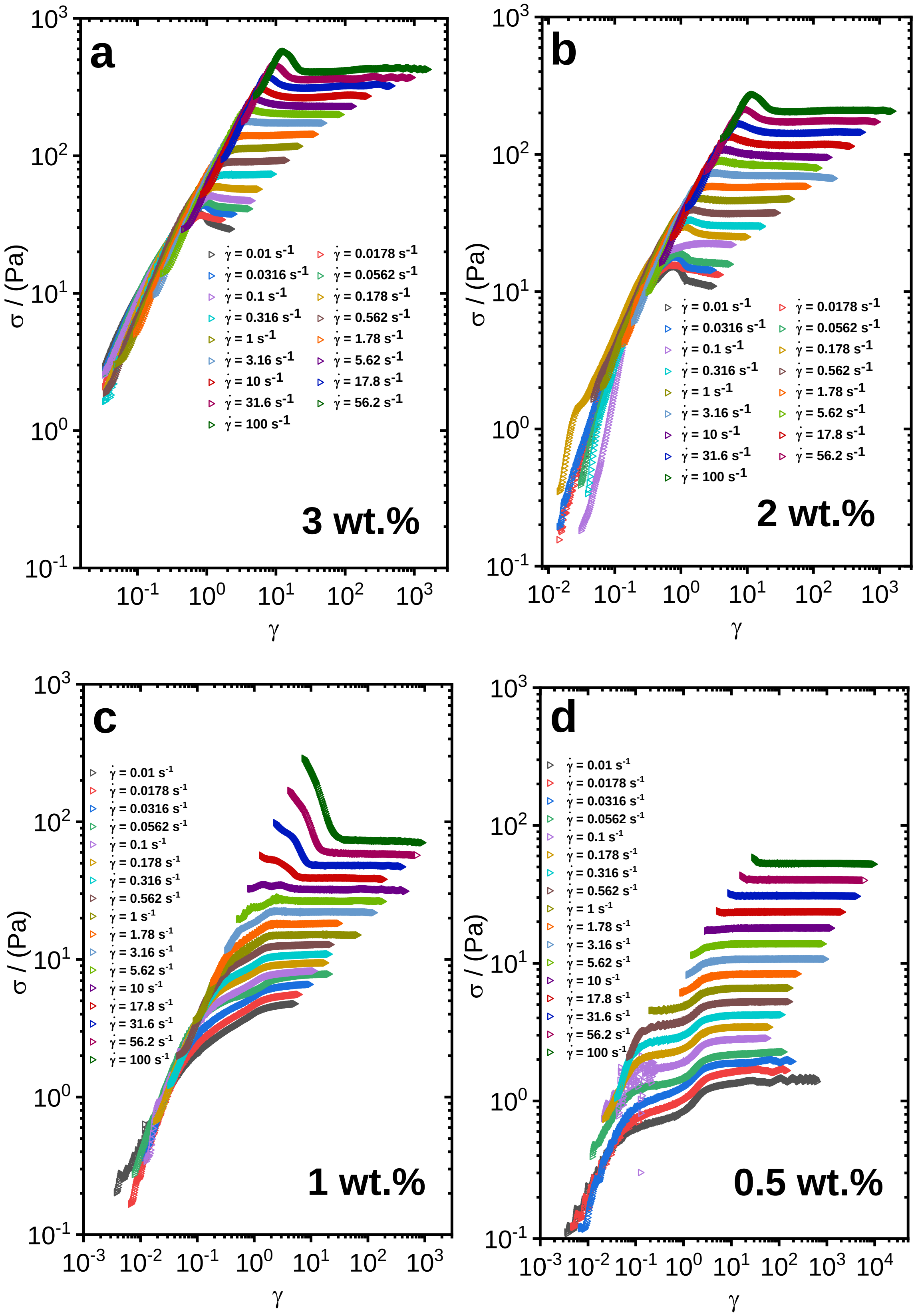
a
b
c
d
3 wt.%
2 wt.%
1 wt.%
0.5 wt.%
σ / (Pa)
γ

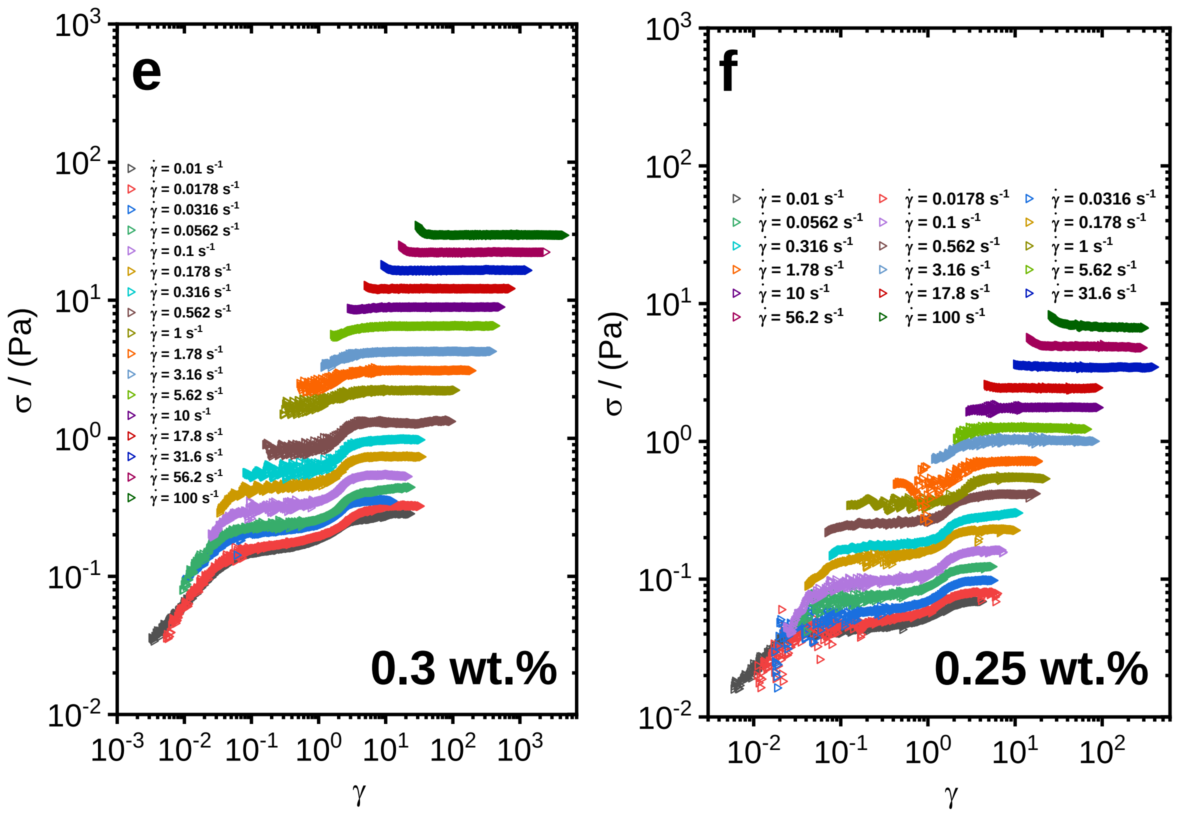

Figure S12. Start-up of the shear rate in terms of the stress response as a function of strain at various shear rates (reported in the legend) for multiarmed PS-PMAA polyelectrolyte solution at (a) 3 wt.%, (b) 2 wt.%, (c) 1 wt.%, (d) 0.5 wt.%, (e) 0.3 wt.%, and (f) 0.25 wt.%.
